# Advances in Nanotheranostic Systems for Concurrent Cancer Imaging and Therapy: An Overview of the Last 5 Years

**DOI:** 10.3390/molecules29245985

**Published:** 2024-12-19

**Authors:** Anna Małgorzata Lankoff, Malwina Czerwińska, Marcin Kruszewski

**Affiliations:** 1 Centre for Radiobiology and Biological Dosimetry, Institute of Nuclear Chemistry and Technology, Dorodna 16, 03-195 Warsaw, Poland; m.kruszewski@ichtj.waw.pl; 2 Department of Medical Biology, Institute of Biology, Jan Kochanowski University, Uniwersytecka 15, 25-406 Kielce, Poland; 3 Department of Dietetics, Institute of Human Nutrition Sciences, Warsaw University of Life Sciences (WULS-SGGW), 159c Nowoursynowska Str, 02-776 Warsaw, Poland; m.czerwinska@ichtj.waw.pl; 4 Department of Molecular Biology and Translational Research, Institute of Rural Health, Jaczewskiego 2, 20-090 Lublin, Poland

**Keywords:** nanotheranostics, nanoparticles, cancer diagnosis, cancer therapy

## Abstract

The rapid development of nanotechnology during the last two decades has created new opportunities to design and generate more advanced nanotheranostics with diversified capabilities for diagnosis, drug delivery, and treatment response monitoring in a single platform. To date, several approaches have been employed in order to develop nanotheranostics. The purpose of this review is to briefly discuss the key components of nanotheranostic systems, to present the conventional and upcoming imaging and therapeutic modalities that employ nanotheranostic systems, and to evaluate recent progress in the field of cancer nanotheranostic systems in the past five years (2020–2024). Special attention is focused on the design of cancer nanotheranostic systems, their composition, specificity, potential for multimodal imaging and therapy, and in vitro and in vivo characterization.

## 1. Introduction

The word “theranostic” is a hybrid term made by fusion of two words, therapeutic and diagnostic, thus referring to a combination of diagnosis and treatment. This term was used for the first time in 1998 by John Funkhouser, who developed a test for monitoring the efficacy of a new anticoagulant drug [1]. However, the concept of theranostics has been clinically adopted in nuclear medicine for over 80 years, starting when Saul Hertz applied radioactive iodine (I-131) for the treatment of patients with hyperthyroidism for the first time in early 1941 [2]. Thanks to this pioneering work of Hertz, the first theranostic procedure with I-131 was used in the mid-1950s for the diagnostic scintigraphy and therapy of thyroid carcinoma [3]. Since then, the development of cancer theranostics in nuclear medicine has seen substantial progress [4]. The rapid development of nanotechnology during the last two decades has created new opportunities to design and generate advanced nanotheranostics with diversified capabilities for diagnosis, drug delivery, and treatment response monitoring in a single platform. To date, several approaches have been employed in order to develop nanotheranostics.

This review focuses on the development of cancer nanotheranostic systems in the past five years, reported from 2020 to 2024. First, we shed light on the key components of nanotheranostic systems. Then, we give a brief overview of the conventional and upcoming imaging and therapeutic modalities that employ the nanotheranostic approach. Lastly, we present in detail selected cancer nanotheranostic systems, along with their structure and capabilities in terms of both therapeutic and imaging aspects.

## 2. Key Components of Nanotheranostic Systems

**Figure 1:**
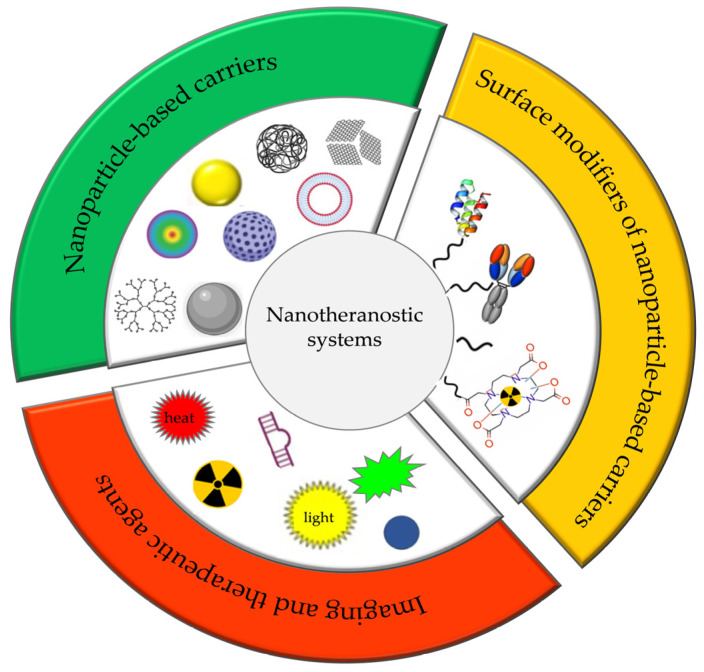
The main components of nanotheranostic systems, including a nanoparticle-based carrier, surface modifiers, and biomedical payload.

The main components of nanotheranostic systems include at least three elements: a nanoparticle-based carrier, surface modifiers, and biomedical payload [5] (Figure 1).

### 2.1. Characteristics of Ideal Nanoparticle-Based Carriers for Nanotheranostic Systems

So far, a variety of nanoparticle-based carriers have been proposed and tested for nanotheranostic applications, such as metal and metal oxide nanoparticles (iron oxide, gold and silver), polymers, silica nanoparticles, liposomes, dendrimers, carbon-based nanoparticles, quantum dots, and composite nanoparticles [6,7,8,9]. Nanoparticle-based carriers should fulfill several important requirements to create an ideal nanotheranostic system. First of all, they should be characterized by high drug loading content and efficiency to encapsulate target molecules and diagnostic agents. Drug loading content and efficiency are determined by both the nanocarrier and payload properties [10]. The most important properties of nanocarriers that affect drug loading content and efficiency include their tiny size, with at least one dimension in the 1–100 nanometer range, surface-to-volume ratio, surface charge, porous structure, composition, and the possibility of introducing functional groups on both external and internal porous surfaces, as well as mechanical, chemical, biological and thermal stability. The payload properties that influence loading content and efficiency include their molecular weight and net charge, concentration in solution, solubility in a nanocarrier, diffusion limitations, ionic interactions, and also chemical interactions between the payload and nanocarrier [11]. In addition, the nanocarrier should be stable enough to protect the payload from degradation, allow drugs to circulate in the bloodstream for longer periods, and provide precise and controlled release of cargo in cancer cells [12]. In order to enable such controlled release of drugs, stimuli-responsive nanocarriers are usually made of a hydrophobic inner core and a hydrophilic or amphiphilic outer shell, which is an amphiphilic stimulus-responsive polymer sensitive to various endogenous or exogenous stimuli. They can also contain various environmentally sensitive functionalities within their structures, like stoppers that seal the internal pores of nanocarriers or spacers used for the conjugation of cargo to nanocarriers. The disruption of these structures and further payload release may be triggered by external stimuli (e.g., light, temperature, ultra-sound, magnetic field, etc.) or by changes in the cellular environment (e.g., pH, enzymatic, or redox reactions) [13]. The dynamics of this process depend on many factors, such as the size of the nanocarrier, surface charge, and porosity, and are influenced by several mechanisms, including drug dissolution from the nanocarrier surface, diffusion through the nanocarrier matrix, and degradation or erosion of the nanocarrier matrix over time. Apart from these essential requirements, nanocarriers should possess other unique properties, such as high labeling efficiency and a high degree of modification and functionalization with target ligands. Thanks to the possibility of modifying and functionalizing the surface of nanocarriers, it is possible not only to target the delivery of drugs and/or contrast agents to cancer cells but also to improve their biocompatibility and reduce their toxicity. Biocompatibility refers to the ability of a biomaterial to perform its desired function with respect to a medical therapy without eliciting any undesirable interactions with the host tissues and cells in a specific situation [14]. The most obvious indication of such interaction is the cellular response, which might be considered curative, neutral, or toxic. The toxicity of nanocarriers is a serious problem and health risk [15,16]. Growing evidence from in vitro and in vivo studies indicates that the possible short- or long-term deposition of various nanocarriers in body tissues may lead to cytotoxicity, immunotoxicity, genotoxicity, carcinogenicity, reproductive toxicity, systemic toxicity, and others [17]. Therefore, before the clinical application of nanocarriers in nanotheranostic systems, these risks must be clearly addressed through adequate demonstration of their biosafety in vivo.

### 2.2. Surface Modifiers of Nanoparticle-Based Carriers

The second element of nanotheranostic systems are the surface modifiers, which offer numerous advantages, including the enhanced biocompatibility of nanoparticle-based delivery systems, high cellular internalization capability, efficient intracellular distribution, colloidal stability, regulated assembly, and target specificity. Since the mechanisms of nanoparticle surface modification for biomedical applications were reviewed elsewhere in detail [18,19], here, we give only a general overview of this issue. In the initial phase of surface modification, homo- or hetero-bifunctional crosslinkers are employed to introduce a variety of organic functional groups. For instance, the surface of silica nanoparticles can be modified utilizing aminosilanes, which impart amino groups that are beneficial for subsequent conjugation with the compound of interest [20]. The surface of gold nanoparticles can be modified through the application of crosslinkers bearing -SH or -NH_2_ groups, which facilitate the formation of covalent bonds with the metal [21]. Additionally, the surfaces of liposomes or micelles can be modified by the integration of polyethylene glycol (PEG) to lipid anchors [22]. In the context of metal oxides and quantum dots, the predominant strategy involves substituting the original chemical moieties present on the nanoparticle’s surface with functional groups of interest, such as amines, carboxylic acid groups, diols, or thiols [23]. These functional groups may subsequently be conjugated with chitosan, dextran, polyethyleneimine, mono- or bifunctional PEG molecules, silicon, catechol, polymers, lipid and lipopolymer coatings, amino groups, spacers, mono- or bifunctional chelators, radionuclides, or receptor-targeting ligands [24].

### 2.3. Imaging and Therapeutic Agents in Nanotheranostic Systems

Imaging and therapeutic agents in nanotheranostic systems enable smarter, more personalized, and more effective cancer treatment. The efficacy of the imaging and therapeutic agents in nanotheranostic systems considered in this review is described in detail in Section 4.

#### 2.3.1. Imaging Agents in Nanotheranostic Systems

The incorporation of imaging agents into nanotheranostics offers significant benefits, enhancing both diagnostic and therapeutic capabilities. Imaging agents facilitate the non-invasive assessment of the biodistribution and accumulation of drugs and their release at the target site. They also allow for the monitoring of treatment responses, which helps with proper therapy adjustment, leading to better patient management. Moreover, some of them allow for tumor visualization and monitoring based on molecular signatures in real time. To date, numerous imaging agents have been used in nanotheranostic systems for the detection of cancer by magnetic resonance imaging (MRI), computed tomography (CT), single-photon emission computed tomography (SPECT), positron emission tomography (PET), photoacoustic imaging (PAI), and optical imaging, including fluorescence imaging (FI) and near-infrared (NIR) imaging, as well as FRET imaging.

MRI imaging agents are widely used in nanotheranostics for high-resolution, non-invasive visualization of anatomical structures and physiological processes. The use of MR contrast agents enhances the MRI signal, improving the visibility of specific tissues, tumors, and vascular structures. The most frequently used contrast agents in nanotheranostics for MRI include type T2-weighted contrast agents (negative), such as superparamagnetic iron oxide nanoparticles (SPIONs) and ferrites (zinc ferrite ZnFe_2_O_4_ and manganese ferrite MnFe_2_O_4_); type T1-weighted contrast agents (positive), such as gadolinium (Gd^3+^) and manganese (Mn^2+^); and type T1- or T2-weighted contrast agents, such as europium oxide (Eu_2_O_3_), dysprosium oxide (Dy_2_O_3_), and terbium (Tb). CT imaging agents increase the density of specific tissues, enhancing image clarity. They increase X-ray absorption and provide strong contrast in CT images. The most frequently used contrast agents in nanotheranostics for CT include gold, tungsten, bismuth, iodine, and barium nanoparticles. SPECT contrast agents (also called tracers or radiotracers) are gamma-emitting radionuclides which allow for the generation of 3D images of biological tissues with high sensitivity. They can track nanoparticles in real time and facilitate tumor imaging, drug delivery tracking, and treatment monitoring. The PET radionuclides most frequently used in nanotheranostics include technetium-99m [^99m^Tc], gallium-67 [^67^Ga], indium-111 [^111^In], and iodine-123 [^123^I]. PET agents are positron-emitting radionuclides, which provide real-time, quantitative information about biological processes in the body. They can track the biodistribution of nanotheranostic systems and their accumulation and clearance in vivo. Moreover, PET radionuclides allow for real-time tracking of drug delivery and release, as well as enabling the precise imaging of tumors and metastatic clusters. The most frequently used PET radionuclides in nanotheranostics include galium-68 [^68^Ga], zirconium-89 [^89^Zr], copper-64 [^64^Cu], and fluorine-8 [^8^F]. PAI agents allow for strong absorption in NIR (700–1100 nm), high photothermal conversion efficiency, and high thermal expansion. The most frequently used PAI agents in nanotheranostics include indocyanine green (ICG) (FDA-approved NIR dye with strong absorption at 800 nm), gold nanoparticles, carbon-based nanoparticles (carbon nanotubes and graphene oxide), molybdenum disulfide (MoS_2_) nanoparticles, and copper sulfide (CuS) nanoparticles. Optical agents include agents for fluorescent imaging (FI) and for NIR imaging. Optical imaging agents are powerful tools for not only disease diagnosis but also for following disease progression and treatment monitoring at the cellular and molecular levels. Moreover, they allow for tumor visualization and monitoring in real time, enabling fluorescence-guided surgery during the resection of cancer. This helps in delineating the margins of the tumor, which can often be invisible/unclear. The optical imaging agents that are currently approved for use in the clinic or are in clinical trials and under investigation for nanotheranostic applications include FI agents (e.g., Cy5 fluorophore, Cy5.5 fluorophore, IRDye 800CW fluorophore, AlexaFluor 647 fluorophore), NIR-I agents (e.g., indocyanine green (ICG), rare-earth-doped materials like NaYF_4_:Yb/Er, carbon dots, graphene quantum dots, copper sulfide (CuS) and copper oxide (CuO) nanoparticles, gold nanoparticles, Cy5.5 fluorophore, Cy7 fluorophore, AlexaFluor 750 fluorophore) and NIR-II agents (e.g., organic IR-1061, IR-1048, IR-1051, CH1055 dyes, gold nanoparticles, Ag quantum dots, Er^3+^-, Tm^3+^-, and Nd^3+^-doped nanoparticles). FRET imaging agents include two chromophores, which create a donor–acceptor pair with a distance of 1-10 nm. They provide the ability to visualize cellular processes, track drug release, and detect enzymatic activity. These agents also enable image-guided therapy. The most frequently used FRET agents in nanotheranostics include fluorophores, such as fluorescein (donor)–rhodamine (acceptor), Cy3 (donor)–Cy5 (acceptor), Alexa 488 (donor)–Alexa 594 (acceptor), quantum dots as donor–acceptor pairs, and lanthanide Eu^3+^ or Tb^3+^ (donor)–fluorophore or quantum dots (acceptor).

#### 2.3.2. Therapeutic Agents in Nanotheranostic Systems

Therapeutic agents in nanotheranostic systems may include chemotherapy (CTX) agents, radiotherapy (RT) agents, photodynamic therapy (PDT) agents, photothermal therapy (PTT) agents, boron neutron capture therapy (BNCT) agents, and high-intensity focused ultrasound (HIFU) agents. The most frequently used chemotherapeutics in nanotheranostic systems include small-molecule drugs such as doxorubicin, paclitaxel, cisplatin, curcumin, palbociclib, chlorambucil, baicalina, and chalcone. The group of radiotherapy agents included radionuclides (e.g., iodine-131 [^131^I], lutetium-177 [^177^Lu], actinium-225 [^22^⁵Ac], indium-111 [^111^In]) and radiotherapy-sensitizing agents such as gold and hafnium oxide nanoparticles. The most frequently used agents for photodynamic therapy (PDT) are porphyrins, chlorin e6, YC-9, iridium complex Ir1, and indocyanine green (ICG). Photothermal therapy (PTT) agents in nanotheranostic systems mainly include gold nanoparticles, carbon-based nanoparticles, semiconductor nanoparticles (CuS, MoS_2_), and polydopamine. Boron-containing peptides and proteins (e.g., carboranes) and boron-containing nucleosides and nucleotides (e.g., boronated deoxyuridine) are the most frequently used agents for BNCT. Moreover, therapeutic agents may also include DNA (e.g., anti-miR-21 oligonucleotide or plasmid DNA-encoding pDNA-HIC1), agents which inhibit protein expression at the RNA level, and proteins/peptides (enzymes and antibodies) which inhibit different signaling pathways (e.g., the PI3K/mTOR signaling pathway).

## 3. Conventional and Upcoming Imaging and Therapeutic Modalities Enabled by Nanotheranostic Systems

Nanotheranostics integrates diagnostic imaging and therapeutic strategies into a single platform, enabling real-time image-guided drug delivery and multimodal imaging, as well as ensuring the spatial and temporal alignment of imaging and the selective visualization of cancer tissues, followed by the release/activation of therapeutic molecules, multimodal imaging-guided therapy, and personalized treatment. To achieve this seamless integration, it is crucial to establish effective transitions between imaging and therapy which ensure that the diagnostic feedback guides therapeutic interventions with precision. Strengthening the transitions between imaging modalities and therapeutic strategies requires an approach involving stimuli-responsive nanocarriers, multimodal imaging, and real-time feedback mechanisms. Advances in dual/multimodal imaging systems, stimuli-responsive nanotheranostics, and multifunctional nanocarriers ensure seamless switching from diagnosis to therapy. By integrating imaging, drug delivery, and therapeutic intervention, nanotheranostics paves the way for more personalized and effective cancer treatments.

### 3.1. Conventional and Upcoming Imaging Modalities

A variety of conventional and upcoming imaging strategies have been used in nanotheranostic systems over the past years; these include MRI, CT, USI, SPECT, PET, PAI, FI, NIFR I/II imaging, and FTIR. The advantages and limitations of these imaging strategies are summarized in Supplementary Table S1.

MRI is one of the most accurate non-invasive imaging modalities. It requires a strong and uniform magnetic field to generate high-resolution 3D images of soft tissues with adaptable image contrast parameters. There are two categories of MRI: T1-weighted and T2-weighted MRI. T1-weighted MRI decreases the signal of water and increases the signal of fatty tissue, whereas T2-weighted MRI increases the signal of water [25]. MRI possesses unique features, including no penetration depth limitation, excellent spatial (1–2 mm) and temporal (minutes to hours) resolution, and safe operation. However, due to its low sensitivity and the long time needed to obtain the images, contrast agents (described in Section 2.3.1) are used to enhance its signals. These agents increase signal intensity on T1-weighted images or reduce signal intensity on T2-weighted images by shortening the T1 or T2 relaxation time. The contrast efficiency of these agents is quantified by the longitudinal relaxation (r_1_) and transverse relaxation (r_2_) values and by the r_2_/r_1_ ratio. The higher the r_2_/r_1_ ratio, the greater the efficiency of a T2 contrast agent and vice versa for a T1 contrast agent [26].

CT imaging is a three-dimensional, computerized X-ray imaging procedure that allow for a clearer view by stacking cross-sectional images. Because tissues do not show contrast to each other, contrast agents are used to improve spatial resolution [27].

SPECT is a nuclear imaging modality using gamma rays. SPECT produces 3D images of the distribution of a radioactive tracer which allow for assessing the perfusion and functionality of specific tissues. SPECT has high sensitivity and provides dynamic tracking imaging but has lower spatial resolution than MRI. The main disadvantage of routine SPECT is the lack of anatomic information [28].

PET is a functional, non-invasive nuclear imaging modality which provides 3D images with high sensitivity, based on the ability of a scanner to detect positrons from various positron-emitting radioisotopes. These radioisotopes enable the functional imaging of several metabolic processes, blood flow measurements, regional chemical composition, and/or chemical absorption [29].

PAI is a high-contrast spectroscopy based on optical imaging with high spatial resolution. PAI provides anatomical, functional, and molecular information. The clinical application of PAI involves the use of photoacoustic imaging as a tool to support photothermal therapy. PAI uses tissue chromophores that absorb incident light to generate acoustic waves in the ultrasonic range. The acoustic waves are detected by traditional ultrasound and cross-sectional scanners. Finally, 3D images are reconstructed from the acoustic waves [30]. PAI possesses several advantages as compared to other optical or ultrasound imaging techniques: (1) it is a multi-scale and multi-contrast imaging modality, (2) it provides longer penetration depths in soft tissues compared to pure optical methods, (3) it provides high-quality in vivo images, (4) it generates no speckle artifacts, (4) PAI systems are less expensive as compared with MRI and PET [31].

Fluorescence imaging (FI) is categorized as visible (400–700 nm), first near-infrared (NIR-I, 700–900), and second near-infrared (NIR-II, 1000–1700 nm) [32].

NIR-I fluorescence imaging has gained significant attention in biomedical research due to its high sensitivity, prompt feedback, lack of hazardous radiation, and relatively low cost. NIR-I fluorophores have been widely used for the real-time imaging of tumor cells and their clusters and intraoperative image-guided surgical removal of lymph nodes and tumor tissues [33].

NIR-II fluorescence imaging is the most advanced imaging fidelity method with extraordinary penetration depth, surpassing that of visible and NIR-I probes, signal-to-background ratio, biocompatibility, and targeting ability. Typically, the pronounced absorption by NIR-II fluorescent material at or above 800 nm suggests its application for PTT and PAT. NIR-II is being widely applied for tumor diagnosis and for fluorescence-imaging-guided tumor surgery [34].

FRET imaging is a specific technique which can be used for studying a wide range of biomedical applications, such as the imaging of biopsy tissues and the imaging of live and fixed cells. FRET imaging gives information on the biochemical status of the cells [35].

### 3.2. Conventional and Upcoming Therapeutic Modalities

Nanotheranostics combine well with conventional and upcoming therapeutic modalities, including chemotherapy (CTX), radiotherapy (RT), photodynamic therapy (PDT), photothermal therapy (PTT), boron neutron capture therapy (BNCT), and high-intensity focused ultrasound (HIFU).

CTX is a systemic cancer treatment that involves the administration of chemotherapeutic agents to inhibit tumor growth at several levels within the cell and finally destroy cancer cells. A plethora of chemotherapeutic agents and natural medicinal anti-cancer compounds which can suppress tumor growth through diverse mechanisms have been devised over the years. Although growing evidence shows that systematic or targeted approaches could be the future of cancer medicine, conventional chemotherapy remains an often-chosen therapeutic option despite its known side effects on the patient’s physical and psychological health [36]. The problems with “off-target” drug delivery can be bypassed by nanotheranostic systems.

RT is a common treatment modality for cancer, useful in about 60% of all cancers. It is a local treatment which uses radiation beams such as photons, protons and electrons. New RT treatment methods, such as 3-D conformal radiation therapy, intensity-modulated radiation therapy (IMRT), image-guided radiation therapy (IGRT), Tomotherapy^®^, and stereotactic body radiation therapy, as well as different modes of delivering the total radiation dose, such as accelerated fractionation, hyperfractionation, or hypofractionation, have improved the accuracy and success of radiation therapy for cancer treatment [37]. Although these technologies have greatly improved the therapeutic outcomes of RT, there are still some challenges with applying RT alone. These problems can be overcome with radiosensitizers, such as functionalized noble metal nanomaterials or nanotheranostics [38].

PDT is a two-stage treatment that combines three basic elements: oxygen, a photosensitizer drug, and light with specific wavelength (usually 660 nm or 880 nm). So far, several photosensitizers have been encapsulated in nanotherapeutic systems; examples include chlorin e6 (Ce6), porphyrins, hematoporphyrin derivatives (Hp), methylene blue (MB), phthalocyanine (Pcs), boron dipyrromethene (BODIPY), and indocyanine green (ICG). After the accumulation of a photosensitizer in tumor tissue by light, energy transfer cascades are switched down, leading to the production of cytotoxic reactive oxygen species (ROS). ROS induce cell death by the oxidation of nucleic acids, proteins, and cellular organelles. Since ROS are only generated in the region irradiated by light, PDT is a low-risk therapy, possessing excellent selectivity, and it may be repeated if necessary [39].

PTT is an emerging therapeutic modality which uses biocompatible carriers with photothermal transduction agents and non-ionizing NIR light to generate heat and locally ablate tumor cells upon irradiation. Photothermal transduction agents commonly used for PTT include metal nanoparticles, such as gold, silver, or platinum, carbon-based materials, such as carbon nanotubes, graphenes, graphene oxides, carbon dots, or MXenes, and other inorganic and organic substances. The absorptions of photothermal transduction agents are usually adjusted to the tissue-transparent window between 750 and 1350 nm, including both the first and second NIR windows. Compared with conventional therapeutic modalities, PTT displays a series of advantages, such as high specificity, minimal invasiveness, and high efficiency [40].

BNCT is a neutron-based radiotherapy that allows for selective cancer treatment at the cellular level. The principle of BNCT relies on the ability of boron to capture thermal neutrons and the assumption that boron-carrying drugs are accumulated selectively in cancer cells [41]. BNCT involves two steps: (1) targeting tumor cells with a non-radioactive boron isotope and (2) exposing boron-containing cells to low-energy neutrons. Capturing a thermal neutron by boron results in the production of 4He^2+^ particles (α particles) and the recoiling of 7Li^3+^ nuclei [42].

HIFU is a novel treatment modality which enables non-invasive tissue heating and ablation [43]. The application of HIFU is limited because of its low spatial resolution and efficacy. However, recently, various multifunctional HIFU synergistic agents have been successfully designed and used in nanotheranostic systems [44].

## 4. In Vitro and In Vivo Studies of Cancer Nanotheranostic Systems

In the past five years from 2020 to 2024, different types of cancer nanotheranostic systems, including metal-based nanotheranostic systems, non-metal-based nanotheranostic systems, core–shell nanoparticles, and nanocomposite hybrids, have been synthesized and evaluated in in vitro and in vivo studies (Table 1).

### 4.1. Metal-Based Cancer Nanotheranostic Systems

Metallic nanoparticles have been used for multiple biomedical applications, such as targeted drug and gene delivery, imaging techniques, radiotherapy enhancement, thermal ablation, antibacterial/antifungal agents, etc. [84]. However, over the last few years, there has been a steadily growing interest in using metallic nanoparticles for theranostic applications [85]. Among them, the most commonly used metallic nanoparticles for theranostic applications include those made of iron, gold, gadolinium, tungsten, bismuth, tungsten, tantalum, ytterbium, silver, platinum, and lead [86].

#### 4.1.1. Iron-Based Nanotheranostic Systems

The most extensively studied iron-based nanoparticles include iron nanoparticles (Fe), iron oxide nanoparticles (magnetite (Fe_3_O_4_) and maghemite (γ-Fe_2_O_3_) or a mixed version of both forms), and ferrites (MFe_2_O_4_, where M = Mn, Fe, Co, Ni, Cu, and Zn) [87,88]. They offer a wide range of desired physical and chemical features, including superparamagnetism, enhanced magnetic susceptibility, high surface-to-volume ratio, high degree of functionalization, high adsorption capacity, good solution stability, high safety, and low cost. All these properties make them ideal candidates for MRI contrast agents, PDT agents, PTT agents, and image-guided drug carriers [89]. In recent years, intense studies have investigated the efficiency of iron-based nanoparticles as theranostic agents; some of them are described below.

**Figure 2:**
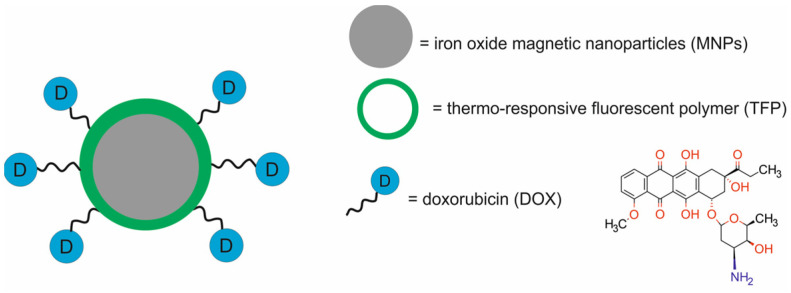
Schematic representation of the DOX-TFP-MNPs nanotheranostic system.

In 2020, Pandey et al. (2020) [45] described the DOX-TFP-MNP multifunctional nanoparticle, composed of an iron oxide magnetic nanoparticle (MNP) coated with a novel thermo-responsive fluorescent polymer (TFP) and loaded with doxorubicin (DOX) for multimodal (optical and MRI) imaging and for chemotherapy (Figure 2). The TFP was synthesized by the copolymerization of poly(N-isopropylacrylamide), allylamine, and a biodegradable photoluminescent polymer.

The DOX-TFP-MNPs possessed excellent colloidal stability and had a degradable shell that eliminated long-term toxicity concerns. The sharp volume phase transition of these nanoparticles allowed for the burst release of DOX, followed by a more sustained release, especially at 41 °C. In vitro studies performed on human dermal fibroblasts (HDFs), normal prostate epithelial cells (PZ-HPV), human skin cancer A431 and G361 cells, and human prostate cancer PC3 and LNCaP cells showed that the TFP-MNPs were not toxic and were taken up by the cells in a dose-dependent manner. Also, the Dox-TFP-MNPs were effective in killing cancer cells under simulated hyperthermia conditions. In vivo imaging studies of DOX-TFP-MNPs in NOD SCID mice bearing PC3-KD xenografts revealed their optical and MR imaging capabilities. Furthermore, in vivo results showed that the DOX-TFP-MNPs, magnetically recruited to the tumor region, delivered therapeutic doses of doxorubicin and inhibited tumor growth.

**Figure 3:**
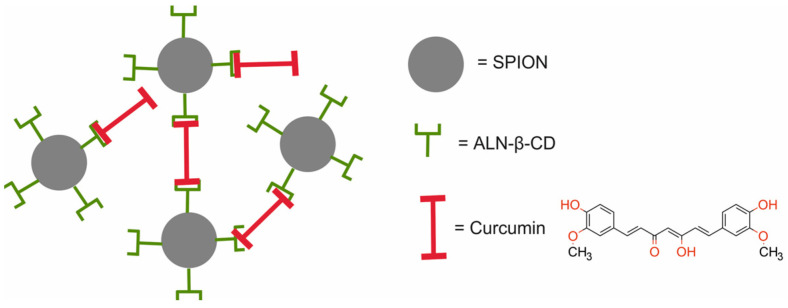
Schematic representation of the Cur/ALN-β-CD-SPIONs nanotheranostic system.

Shen et al. [46] employed the Cur/ALN-β-CD-SPIONs nanotheranostic system, composed of superparamagnetic Fe_3_O_4_ nanoparticles (SPIONs) modified with alendronate-β-cyclodextrin conjugate (ALN-β-CD) and then cross-linked to curcumin (CUR) for chemotherapy and magnetic resonance imaging (MRI) (Figure 3).

In vitro studies demonstrated that the Cur/ALN-β-CD-SPIONs had excellent magnetic and T2 imaging performance and presented a higher saturation of magnetization than that of ALN-β-CD-SPIONs. The r_2_ value of the Cur/ALN-β-CD-SPIONs was two to three folds higher compared with other contrast agents used in the clinic. This was confirmed in vivo in Balb/c mice bearing murine 4T1 xenografts. The results revealed that the Cur/ALN-β-CD-SPIONs showed superior magnetic and T2 imaging performance. Furthermore, mice treated with the Cur/ALN-β-CD-SPIONs displayed a better tumor inhibition effect than that of free curcumin. The mean weight of tumors from the Cur/ALN-β-CD-SPIONs treated mice was 3.1-fold lower than that of the PBS group and 1.34-fold lower than that of mice treated with free curcumin. Moreover, mice receiving the Cur/ALN-β-CD-SPION therapy showed no obvious pathological changes, indicating the safety of the treatment.

**Figure 4:**
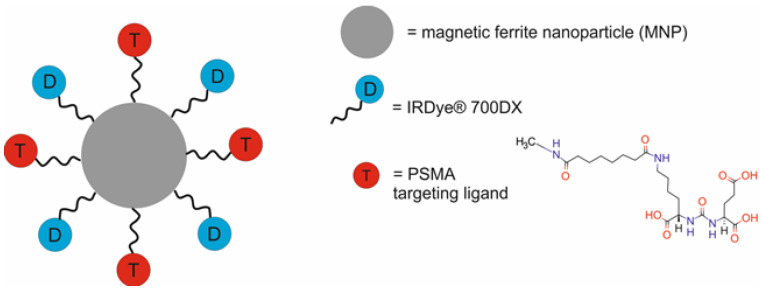
Schematic representation of the YC-9-MNPs nanotheranostic system.

Ngen et al. [47] evaluated the feasibility of the use of a YC-9-MNP nanotheranostic system composed of magnetic ferrite nanoparticles (MNPs) modified with polyethylene glycol (PEG) and functionalized with the PSMA-targeted photosensitizer (YC-9) for targeted PDT and MRI (Figure 4). The YC-9 was synthesized by the conjugation of a near-infrared photosensitizer (IRDye^®^ 700DX) to a low-molecular-weight PSMA ligand (Glu-Lys-urea-suberate((((*S*)-1-carboxy-5-(7-carboxyheptanamido)pentyl)carbamoyl)-l-glutamic acid) by a flexible linker.

In this study, the authors evaluated different PDT pretreatment plans to increase the vascular permeability of PSMA(+) prostate tumors. In vivo experiments were performed in mice bearing PSMA(+) PC3 PIP and PSMA(−) PC3 flu tumors. In vivo fluorescence image-guided PDT revealed a remarkable difference in the accumulation of the YC-9-MNPs in PSMA(+) tumors and PSMA(−) tumors. At the 4 h time point, the YC-9-MNPs accumulated ~3 times more in PSMA(+) tumors than in PSMA(−) tumors and ~17 times more in PSMA(+) tumors than in other regions of the mouse body. However, a marked uptake of the YC-9-MNPs was detected in both PSMA(+) and PSMA(−) tumors 4 h after administration. Furthermore, a high concentration of the YC-9-MNPs was also detected in the bladder, indicating its renal clearance. The in vivo MRI results of early tumor response to PDT demonstrated significant edema in the tissue surrounding the PSMA(+) tumors compared to that surrounding the PSMA(−) tumors. Moreover, the in vivo MRI imaging results revealed a significant increase in the uptake of YC-9-MNPs into the PSMA(+) tumors treated with PDT compared to the PSMA(+) tumors not treated with PDT at 18 h, 42 h, and 66 h post-PDT and YC-9-MNP administration.

**Figure 5:**
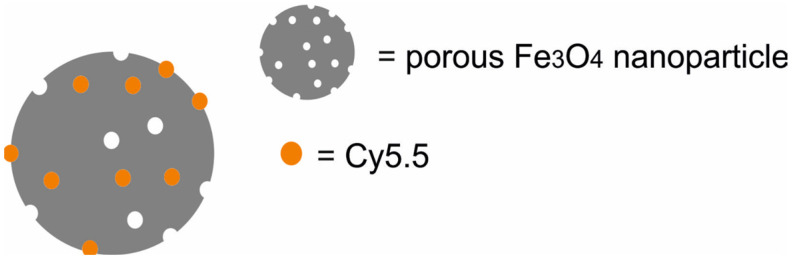
Schematic representation of the Fe_3_O_4_-Cy5.5 nanotheranostic system.

An interesting approach to multimodal MRI/NIFR/PAI imaging and PTT was proposed by Wang et al. [48], who developed a macrophage-mediated Fe_3_O_4_-Cy5.5 photothermal nanoprobe composed of porous Fe_3_O_4_ nanoparticles loaded with Cy5.5 (Figure 5). In vitro studies demonstrated that the Fe_3_O_4_-Cy5.5 nanoparticles had very low toxicity to macrophages and were taken up by macrophages in large quantity without affecting their activity. In vitro PTT indicated that the Fe_3_O_4_-Cy5.5 produced a good killing effect in rat glioma C6 cells under 808 nm laser irradiation. In vivo fluorescence images showed that the brain tumor area of rats bearing C6 glioma xenografts injected with macrophages containing Fe_3_O_4_-Cy5 had obvious fluorescence hyperintensity. Prussian blue staining results showed that a large amount of iron was carried through macrophages and distributed around the tumor tissue through the blood–brain barrier.

In vivo MR experiments confirmed these results and demonstrated that one day after the injection of macrophages containing Fe_3_O_4_-Cy5 into rats bearing C6 glioma xenografts, hypointense shadows showed up around the glioma xenografts and gradually increased 3 days post-injection. On the 5th day after injection, these shadows totally surrounded the glioma xenografts. The boundary of tumors, delineated by in vivo MR/fluorescence imaging, corresponded with histopathological staining. A biodistribution study revealed that 24 h post-injection, the Fe_3_O_4_-Cy5-treated macrophages mainly accumulated in the spleen and kidneys, without causing severe damage to normal organs. PAI revealed that maximal tumor signal was about 10-fold higher relative to that before injection. When the rat heads were subjected to PTT (irradiation with an 808 nm laser for 5 min), tumor volume was significantly reduced on days 3 and 10.

**Figure 6:**
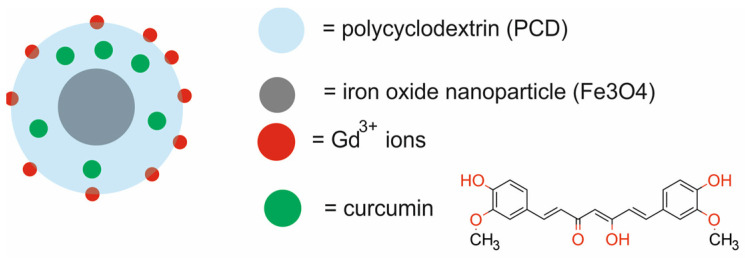
Schematic representation of the Fe_3_O_4_@PCD-Gd/CUR nanotheranostic system.

Mansouri et al. [49] developed a Fe_3_O_4_@PCD-Gd/CUR nanotheranostic system composed of iron oxide nanoparticles (Fe_3_O_4_) coated with a polycyclodextrin (PCD) layer, functionalized with gadolinium ions (Gd), and loaded with curcumin (CUR) for chemotherapy and MRI (Figure 6). The PCD was synthesized by the polymerization of β-cyclodextrin (βCD). Diethylenetriaminepentaacetic dianhydride (DTPA-DA) was used as a cross-linking agent. The βCD in the structure of PCD was a good medium for loading the CUR and further release under controlled conditions. Moreover, the DTPA in the PCD was responsible for selective Gd^3+^ ion chelation. The Gd^3+^ ions served as an enhancer of T1-weighted MRI.

Physicochemical analyses confirmed the good magnetic properties of the Fe_3_O_4_@PCD-Gd NPs as both a T1-weighted and T2-weighted contrast agent in MRI and the high loading efficiency of CUR (88%). The dissolution profile of CUR demonstrated a pH-sensitive release of CUR. In vitro studies revealed that the Fe_3_O_4_@PCD-Gd NPs possessed no significant toxicity against normal and cancerous cell lines (MCF 10A and 4T1). However, CUR-free NPs showed more toxicity against 4T1 than MCF 10A cells. In vivo MRI of BALB/C mice demonstrated contrast enhancement for both T1-positive and T2-negative MRI. An in vivo study also revealed that free CUR and Fe_3_O_4_@PCD-Gd/CUR markedly suppressed tumor growth in Balb/c mice bearing murine 4T1 xenografts as compared to control group. Fe_3_O_4_@PCD-Gd/CUR demonstrated higher tumor suppression than free CUR. Moreover, Fe_3_O_4_@PCD-Gd NPs showed satisfactory capability in tumor shrinking without any adverse effect on body weight. Histopathological analysis of mice indicated no apparent abnormalities and lesions in any of the major organs, confirming no symptoms of tissue toxicity.

**Figure 7:**
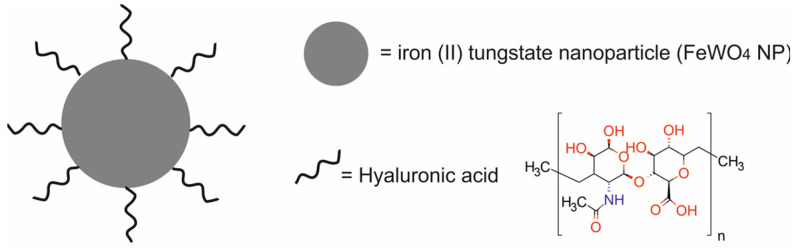
Schematic representation of the HA-FeWO_4_ NPs nanotheranostic system.

In the study described by Yang et al. [50], a HA-FeWO_4_ NP nanotheranostic system was successfully synthesized. It was composed of iron (II) tungstate nanoparticles (FeWO 4 NPs) functionalized with hyaluronic acid (HA) for PTT, MRI, and CT (Figure 7).

The multifunctional HA-FeWO_4_ NPs displayed good dispersion, excellent CT imaging ability, and a strong visible T2WI blackening ability. In vitro studies revealed that the HA-FeWO_4_ NPs exhibited rather low cytotoxicity toward MCF-10A and 4T1 cells and good biocompatibility. T2-weighted MRI demonstrated the good targeting ability and cellular imaging ability of the HA-FeWO_4_ NPs toward 4T1 cells. In vivo studies in Balb/c mice revealed no death, no drop in body weight, and no obvious liver and kidney damage after treatment with HA-FeWO_4_ NPs. Furthermore, in vivo MR/CT dual-modality imaging demonstrated that tumors were clearly visualized by the HA-FeWO_4_ NPs. More importantly, the HA-FeWO_4_ NPs showed excellent photothermal efficiency and tumor inhibitory activity via the hyperthermia killing of cancer cells.

#### 4.1.2. Gold-Based Cancer Nanotheranostic Systems

Gold-based nanoparticles have been extensively studied within biomedicine due to their low toxicity, adjustable size and shape, easy surface functionalization, ability to interact with light via surface plasma resonance, ability to convert optical energy into heat, formation of local density of states, and short-range translational order [90,91]. These unique properties of gold-based nanoparticles make them promising candidates for a wide range of applications, including as contrast agents for PTT, CT, MRI, FI, and PAI, as sensitizing agents for RT, as carriers for the delivery of photosensitizer agents in PDT, and as nanotheranostic systems [92]. The major obstacles that have been faced by gold-based nanotheranostic systems are poor specific targeting and resolution and photostability during PTT.

**Figure 8:**
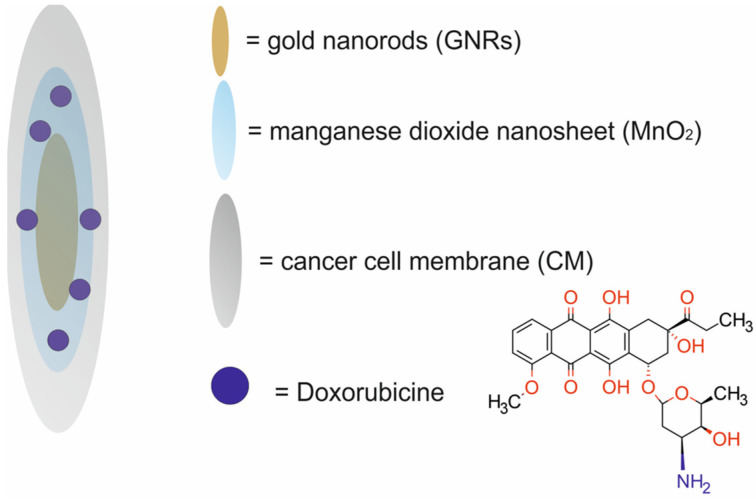
Schematic representation of the CM-DOX-GMNPs nanotheranostic system.

Zhang et al. [51] designed CM-DOX-GMNPs, smart, two-dimensional (2D) supraparticles composed of gold nanorods (GNRs) coated with a manganese dioxide nanosheet (MnO_2_) and cancer cell membrane (CM) and loaded with doxorubicin (DOX) for tumor-targeted MRI/PTI and chemo/PTT (Figure 8). The cell membrane fragments were extracted from 4T1 breast cancer cells and then extruded together with the nanorods coated with MnO_2_ nanosheets through a filter giving a typical core–shell structure with the cell membrane as the shell.

In vitro studies revealed that the MnO_2_ nanosheets on the gold nanorods’ surface were successfully etched by endogenous GSH or acidic H_2_O_2_ to Mn^2+^, which led to the stimuli-controlled release of DOX. The etched Mn^2+^ within the tumor was further used as an MRI contrast agent for imaging. Due to the efficient photothermal conversion effect from gold nanorods in the presence of laser, photothermal therapy was activated for synergistic cancer treatment with DOX. In vitro studies of the CM-DOX-GMNPs revealed their fast cellular internalization, good biocompatibility, drug-releasing property, and antiproliferative effect in mouse breast cancer 4T1 cells. In vivo experiments carried out in Balb/c mice bearing murine 4T1 xenografts illustrated that the CM-DOX-GMNPs displayed tumor-specific MRI/PTI ability and an excellent inhibition effect on tumor growth.

**Figure 9:**
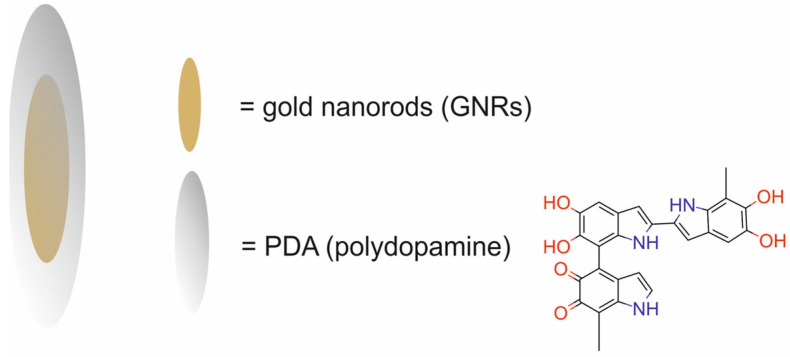
Schematic representation of the GNR@PDA nanotheranostic system.

Yim et al. [52] investigated a GNR@PDA ultra-small nanotheranostic system composed of gold nanorods (GNRs) modified with PEG and coated with polydopamine (PDA) for photoacoustic imaging (PI) and PTT (Figure 9).

The GNR@PDAs showed outstanding conversion stability in four successive cycles, which confirmed its good photostability in the NIR-II window. Furthermore, the GNR@PDAs demonstrated no disassembly of the PDA coating or no morphology changes upon four cycles of laser irradiation. In vitro experiments demonstrated that the GNR@PDA in the NIR-II window showed 95% ablation of human ovarian adenocarcinoma SKOV3 cells, which was significantly higher than that of the large LGNRs (66%) and the GNRs (74%) without PDA surface. These results also demonstrated that the NIR-II laser alone did not cause any cell death. Furthermore, the photothermal performance of gold nanorods was significantly improved when PDA was used as a coating shell.

**Figure 10:**
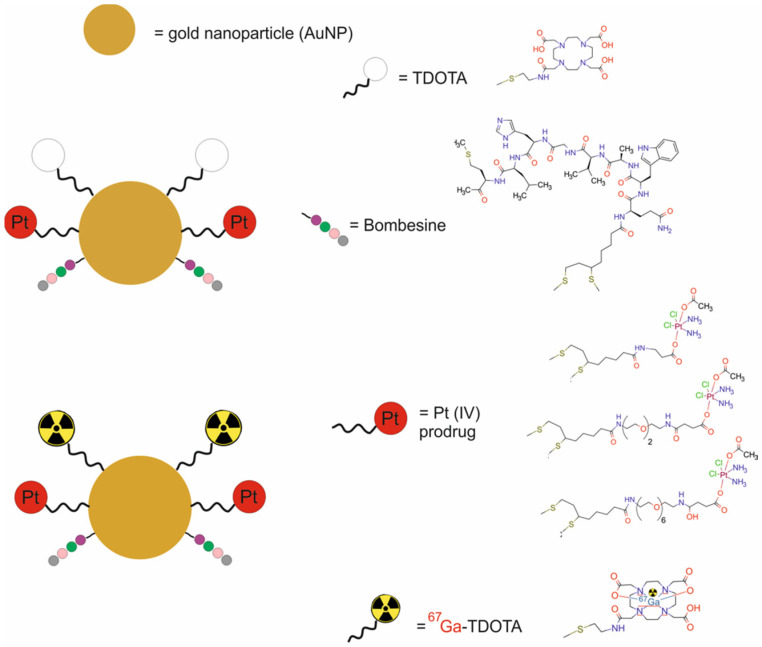
Schematic representation of the ^67^Ga-AuNP-BBN-Pt1 nanotheranostic system.

An active targeting nanotheranostic system was reported by Silva et al. [53], who synthesized ^67^Ga-AuNP-BBN-Pt1, composed of small-core gold nanoparticles (AuNPs) modified with a thiolated DOTA derivative and functionalized with bombesin analog (BBN [7,8,9,10,11,12,13,14]) and a Pt(IV) prodrug. These molecules were attached to the AuNPs without (AuNP-BBN-Pt1) or with a PEGylated linker (AuNP-BBN-Pt2 and AuNP-BBN-Pt3) and radiolabeled with ^67^Ga for SPECT and chemotherapy (Figure 10).

The ^67^Ga-AuNP-BBN-Pt1 displayed good stability after 24 h of incubation with cell medium or apotransferrin. An in vitro study revealed that the ^67^Ga-labeled AuNPs presented fast kinetics of cellular uptake and extensive internalization into the tumor PC3 cells. In the GRPR+ prostate cancer PC3 cell line, the cytotoxic activity of the AuNP-BBN-Pt nanoparticles was strongly dependent on the presence of the PEGylated linker. The AuNP-BBN-Pt1 was much more toxic against the tumor PC3 cell line than against the non-cancerous RWPE-1 cells. In vivo biodistribution results after intratumoral administration of the ^67^Ga-AuNP-BBN-Pt1 in Balb/c-Nude mice bearing PC3 xenografts showed prolonged retention and optimal in vivo stability as compared to cisplatin. Furthermore, microSPECT imaging confirmed the uptake and considerable retention of the ^67^Ga-labeled AuNPs in the tumors.

**Figure 11:**
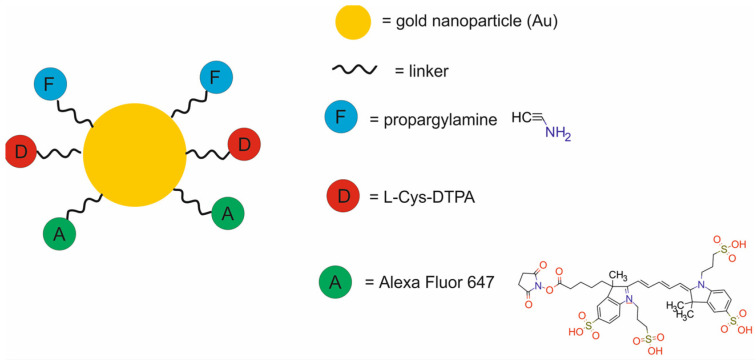
Schematic representation of the Au(L-Cys DTPA) (Propargylamine)-AF647 nanotheranostic system.

Recently, Vogel at al. [54] developed a Au(L-Cys-DTPA)(Propargylamine)–AF647 nanotheranostic system composed of gold nanoparticles (Au) modified with L-Cys-DTPA and propargylamine and labeled with AlexaFluor 647 for fluorescence imaging (FI) and RT (Figure 11). In vitro studies demonstrated that the Au(L-Cys-DTPA)(Propargylamine)–AF647 nanoparticles did not aggregate even over a 24 h period, were readily internalized by rat 9LGS gliosarcoma cells, and produced minimal cytotoxicity. These nanoparticles were simply tracked in vitro thanks to the attached AF647 fluorescent dye. Irradiation of 9LGS cells exposed previously to Au(L-Cys-DTPA)(Propargylamine)-AF647 with 2 and 5 Gy orthovoltage radiation at 125 kVp resulted in a decrease in cell survival.

These nanoparticles also induced DNA damage, as evidenced by the increase in the number of γ-H2AX foci, reflecting double-strand breaks. Finally, these nanoparticles were visible when injected at muscular depth into a Fischer 344 rat cadaver and imaged using optical imaging. The images revealed a significant hot spot at the injection site with a depth of approximately 5 mm.

### 4.2. Non-Metal-Based Cancer Nanotheranostic Systems

Among non-metal-based nanoparticles for cancer nanotheranostic systems, the most studied are polymeric nanoparticles, silica nanoparticles, liposomes, carbon-based nanoparticles, dendrimers, micelles, and nanogels [93]. They are characterized by satisfactory stability and biocompatibility, limited toxicity, favorable drug-loading properties, controlled release and protection of drug molecules within internal and external environment, easy functionalization, and high accumulation in the target tumor tissue [94].

#### 4.2.1. Polymer-Based Cancer Nanotheranostic Systems

Polymer-based nanoparticles have attracted considerable interest over recent years due to their advantageous features, including favorable drug-loading properties, ability to protect drug and other molecules with biological activity against the environment, controlled release of drugs at the tumor site, ease of surface modification and functionalization, biocompatibility, and general non-toxicity [95]. In recent years, considerable efforts have been made to design smart polymer-based nanoplatforms, such as pH- and redox-stimuli-responsive nanoparticles. These features make them a point of interest for the delivery of drugs, proteins, nucleic acids, or imaging/contrast agents to specific tissues or organs [96]. Since polymer-based nanomaterials have been used extensively as carriers for both therapeutic and imaging agents, they hold great promise for the construction of multifunctional nanotheranostic systems [97].

**Figure 12:**
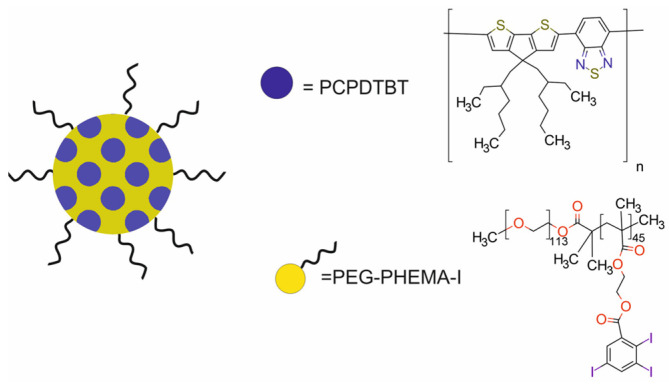
Schematic representation of the SPN-I nanotheranostic system.

One of the multifunctional polymer-based nanotheranostic platforms was the SPN-I semiconducting compound, designed by Zhou et al. [55]. SPN-I was composed of NIR light-absorbing polymer nanoparticles (PCPDTBT) with an iodine-grafted amphiphilic copolymer (PEG-PHEMA-I) for PDT, FI, and CT (Figure 12). The PCPDTBT polymer served as the photosensitizer and source of fluorescence signal, whereas the PEG-PHEMA-I copolymer served as the ^1^O_2_ generation enhancer and nanocarrier.

SPN-I had good colloidal stability after 30-day storage in phosphate-buffered solution. Compared with its counterpart nanoparticles (SPN-Ps), composed of PEG-b-PPG-b-PEG and PCPDTBT, the SPN-I showed 1.5-fold higher ^1^O_2_ generation. Photobleaching studies demonstrated that the SPN-I possessed satisfactory fluorescence intensity and good photostability, even better than that of chlorin e6 (Ce6). In addition, SPN-I presented a high X-ray attenuation coefficient due to the high density of iodine in PEG-PHEMA-I, providing SPN-I with the ability to be used in CT. The results confirmed a linear relationship of CT signal versus the concentration of SPN-I. In vitro studies revealed the effective cellular internalization of SPN-I into Lewis lung carcinoma cells and almost no cytotoxicity without light irradiation, indicating its good biocompatibility. The analysis of the therapeutic efficacy of SPN-I against Lewis cells under 660 nm laser irradiation demonstrated a significant decrease in cell viability. The in vivo application of CT/FI after i.v. injection of SPN-I showed that both CT and FI signal in the tumor area gradually increased over time in C57bl/6 mice bearing Lewis cell xenografts. At 1 h post-injection, the tumor was clearly detected on fluorescence images, while on CT images, it was detected 4 h post-injection. Fluorescence intensity in the tumor area reached maximum at 48 h post-injection. The SPN-I-induced PDT almost inhibited tumor growth in C57bl/6 mice bearing Lewis cell xenografts with a tumor inhibition rate of 98.7%.

**Figure 13:**
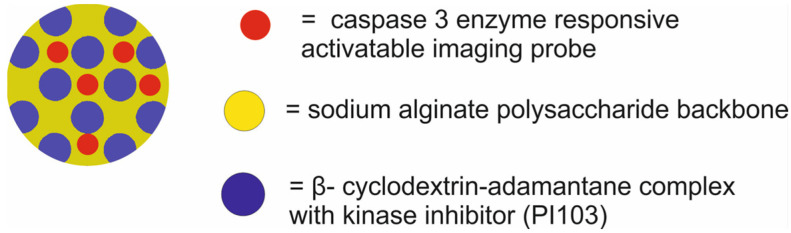
Schematic representation of the SPN nanotheranostic system.

Desphande et al. [56] designed SPNs (supramolecular polysaccharide nanoparticles) composed of a sodium alginate polysaccharide backbone modified with a caspase 3 enzyme responsive activatable imaging probe and loaded with βCD-adamantane complex with a kinase inhibitor (PI103) for therapy by inhibition of the phosphoinositide 3-kinase (PI3K/mTOR) signaling pathway and fluorescence resonance energy transfer imaging (FRET) (Figure 13). The SPNs were constructed by a two-step self-assembly approach. The first stage included synthesis of the activatable probe with a peptide sequence (GK-DEVD-APC) and FRET, which included a dye (5FAM) and a quencher (QSY7) on either side of the peptide sequence. The synthesized probe was then conjugated to the sodium alginate polysaccharide backbone. In the second stage, a stable β-CD-adamantane complex was conjugated with a kinase inhibitor (PI103). Finally, the synthesis of the SPNs was facilitated by the electrostatic interactions between the positively charged β-CD-NH2-complex and the negatively charged sodium alginate backbone.

Physicochemical characterization revealed that the SPNs were very stable in physiologically relevant conditions, whereas they were destabilized in acidic conditions. The results demonstrated the susceptibility of the imaging probe to cleavage under enzymatic conditions. Moreover, the results demonstrated that the release of the caspase-3 enzyme is necessary to monitor the imaging signal. In vitro studies revealed that the SPNs efficiently induced cytotoxic effects and inhibited kinase signaling. The SPNs showed better toxicity than the free kinase inhibitor in triple-negative breast cancer (4T1) cells. In vitro imaging studies confirmed the enzyme-activatable therapy response in cancer cells and in aggressive breast 3D tumor spheroids. In vivo studies on the treatment of C57B/L6 mice bearing D4M xenografts with the SPNs showed significant tumor growth inhibition and therapy response at both early (24-48 h) and late time points. Ex vivo fluorescence imaging confirmed the uptake of the SPNs in the tumor microenvironment.

**Figure 14:**
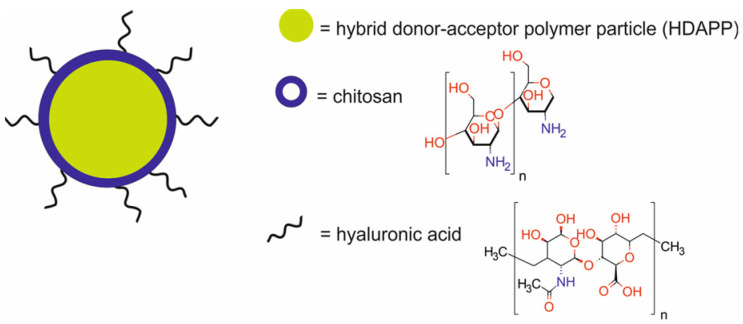
Schematic representation of the HA-HDAPP nanotheranostic system.

McCabe-Lankford et al. [57] developed HA-HDAPPs, hybrid donor–acceptor polymer particles (HDAPPs) modified with chitosan and functionalized with hyaluronic acid (HA) for PTT, FI, and bioluminescence imaging (BI) (Figure 14).

In vitro studies demonstrated that the HA-HDAPPs had a two-fold higher binding to mouse colorectal carcinoma CT26.WT-Fluc-Neo (CT26) cells than unfunctionalized HDAPPs. When cells were exposed to the HA-HDAPPs and stimulated with NIR light, a significant reduction in cell viability was observed. This effect was not present with non-functionalized HA-HDAPPs, which indicates that the binding of the HA-HDAPPs to the cell membrane was critical for photothermal ablation. In vivo photothermal treatment of disseminated colorectal cancer showed that only HA-HDAPPs delivered via intraperitoneal injection reduced the tumor burden. HA-HDAPPs delivered via perfusion together with laser irradiation were not successful in reducing the tumor burden. Moreover, hyaluronic acid-targeted HA-HDAPPs revealed superior binding to tumor regions as compared to untargeted HDAPPs.

**Figure 15:**
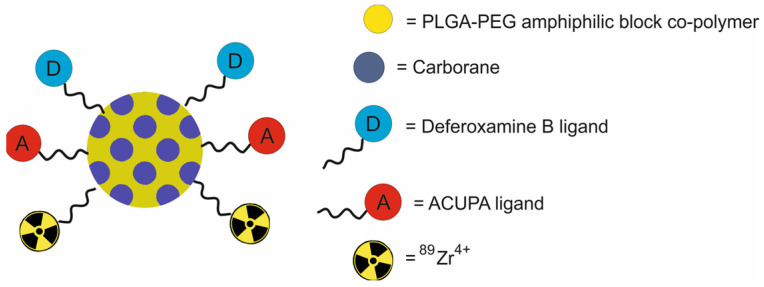
Schematic representation of the [^89^Zr]DFB(25)ACUPA(75) nanotheranostic system.

Meher et al. [58] developed the [^89^Zr]DFB(25)ACUPA(75) nanotheranostic platform, composed of poly(lactide-co-glycolide)-block-poly(ethylene glycol) (PLGA-b-PEG) nanoparticles functionalized with radiometal chelator deferoxamine B (DFB) and the PSMA targeting ligand ACUPA, loaded with the BNCT agent o-carborane, and labeled with ^89^Zr for PET imaging (Figure 15).

In vitro studies demonstrated that the [^89^Zr]DFB(25)ACUPA(75) NPs showed increased PSMA-mediated cell binding and internalization in the metastatic prostate adenocarcinoma PC3-pip cell line transfected with PSMA. However, no evidence of specific binding was observed for the nontargeted [^89^Zr]DFB(25) NPs and the targeted [^89^Zr]DFB(25)ACUPA(25)NPs in the metastatic prostate adenocarcinoma PC3-flu cell line. In vivo μPET/CT imaging in nu/nu athymic mice bearing PC3-flu and PC3-pip xenografts demonstrated high signal in the blood at 2 h, which cleared 20 and 96 h after drug injection. The accumulation of targeted and nontargeted NPs in tumors was below 1% ID/gram. However, a 2-fold higher tumor uptake was obtained in PC3-Pip than in PC3-flu tumors. A high uptake of the [89Zr]DFB(25)ACUPA(75) NPs was observed in the liver and spleen. Biodistribution data at 96 h confirmed that the uptake of the targeted [^89^Zr]DFB(25)ACUPA(75) NPs was around 1%ID uptake/gram in PC3-Pip tumors and was around 0.5%ID uptake/gram in PC3-Flu tumors. However, there was no substantial difference in the accumulation of the targeted [^89^Zr]DFB(25)ACUPA(75) NPs and nontargeted [^89^Zr]DFB(25) NPs in the tumors. In vivo boron biodistribution studies demonstrated little or no targeted boron delivery with [^89^Zr]DFB(25)ACUPA(75).

**Figure 16:**
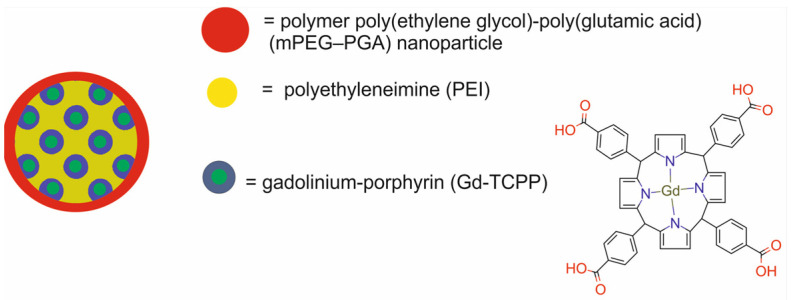
Schematic representation of the Gd–PNP nanotheranostic system.

Chen et al. [59] developed Gd–PNPs polymer poly(ethylene glycol)-poly(glutamic acid) (mPEG–PGA) nanoparticles loaded with gadolinium–porphyrin (Gd-TCPP) and embedded in polyethyleneimine (PEI) for FI, MRI, and PDT (Figure 16). TCPP (4,4′,4″,4‴-(porphine-5,10,15,20-tetrayl) tetrakis (benzoic acid), a typical porphyrin molecule, served a crucial function as the chelating agent for Gd^3+^. The presence of Gd^3+^ on the surface of Gd–PNPs endowed them with MRI capability. The negatively charged Gd–TCPP was mixed with the positively charged supermolecule polyethyleneimine (PEI). The supramolecular Gd–TCPP-PEI was formed by non-covalent, electrostatic interaction-induced self-assembly. Finally, the negatively charged mPEG–PGA polymer was electrostatically adsorbed onto the surface of the Gd–TCPP-PEI.

The results of the Gd^3+^ leakage assay revealed that after 48 h of incubation, only a negligible amount of free Gd^3+^ was released from the Gd–PNPs. Moreover, the Gd-PNPs exhibited good singlet oxygen production property. In vitro studies revealed good biocompatibility of the Gd–PNPs in human ovarian carcinoma Hela cells and mouse colorectal carcinoma CT26 cells, as well as in a zebrafish animal model. In vivo studies confirmed that the Gd–PNPs caused no inflammation and no obvious side effects in all major organs of BALB/c mice, such as the heart, liver, spleen, lungs and kidneys. Dual-modal imaging studies in vitro demonstrated that the cellular uptake process was time-dependent and the Gd–PNPs efficiently accumulated not only in the cell cytoplasm but also in the nucleus. The in vivo FI capability of the Gd–PNPs was confirmed in BALB/c mice bearing murine CT26 xenografts. The results revealed a time-dependent accumulation of Gd–PNPs in tumor tissue. The strongest fluorescent signal was detected 12 h post-intravenous injection and remained after 24 h. Gd–TCPP largely accumulated in the tumor 1 h after injection and diminished by 12 h. Compared to the Gd–TCPP-treated mice, ∼1.6× stronger fluorescent signals were observed in the Gd–PNP-treated mice. Ex vivo fluorescence images of tumors and organs 24 h post-injection demonstrated stronger FL signal from the tumors of Gd–PNP treated mice than from the kidneys, spleen, and liver. In vivo studies also demonstrated that the Gd–PNPs had outstanding T1-MR imaging capability due to their high r1 value. The strongest MR signal with a sharp border of the tumor was observed 6 h post iv.i. In addition, a substantially stronger enhancement effect was observed for the MR signal than for the FL signal. In vitro and in vivo PDT studies after the treatment of cells or mice with the Gd–PNPs under 635 nm laser irradiation confirmed the good tumor cell ablation efficacy of the Gd–PNPs.

**Figure 17:**
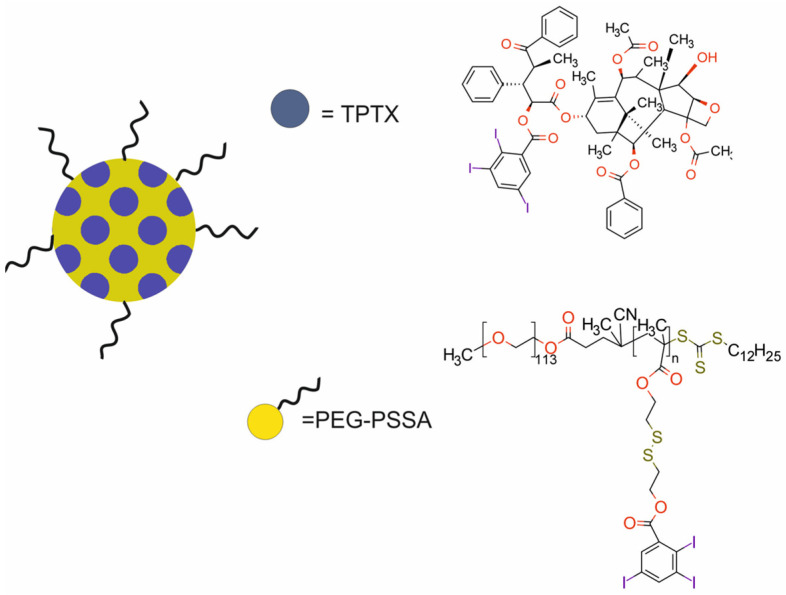
Schematic representation of the SS_TPTX_ nanotheranostic system.

More recently, Chen et al. [60] proposed a SS_TPTX_ nanotheranostic system composed of polymeric poly(ethylene glycol)–poly(styrene sulfonic acid) (PEG-PSSA) nanoparticles loaded with the prodrug TPTX [PTX (paclitaxel)–TIBA (2,3,5-triiodobenzoic acid)] for chemotherapy and CT imaging (Figure 17). TIBA was used to modify PTX and the polymeric vehicle to further increase the encapsulation efficiency of PTX and diminish the systemic toxicity of PTX and TIBA. Physicochemical analyses of SS_TPTX_ demonstrated that more than 90% of the loaded TPTX was released from the SS_TPTX_ within 72 h in the presence of 100 μmol or 10 mmol GSH. In the absence of GSH, approximately 25% TPTX was released from the SS_TPTX_ within 72 h under physiological conditions. Cellular uptake and lysosomal colocalization studies on murine breast cancer 4T1 cells revealed that intracellular fluorescence significantly increased with increasing time. SS_TPTX_ was partially colocalized with lysosomes. Moreover, SS_TPTX_ caused efficient cell cycle arrest and apoptosis like the parent PTX. In vivo studies demonstrated tumor accumulation from 1 to 24 h after injection in Balb/c mice bearing murine 4T1 xenografts, indicating the nanoparticles had accumulated in the tumor and promptly released the loaded drugs in response to the GSH overexpressed in cancer cells. Ex vivo imaging and quantitative analysis of the fluorescence intensity in various organs 24 h post-injection of nanoparticles confirmed the tumor-targeting ability of SS_TPTX_. CT imaging showed that similar concentration-dependent contrast enhancement was observed for SS_TPTX_ and for the commercially available Iohexol. Importantly, SS_TPTX_ revealed a significant tumor growth inhibition effect without any obvious body weight loss at the same time.

**Figure 18:**
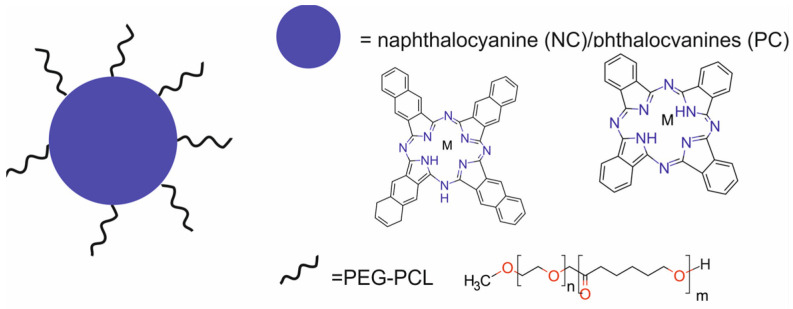
Schematic representation of the CuNC(Octa) nanotheranostic system.

In 2024, Tian et al. [61] reported CuNC(Octa)-loaded micelles, composed of an amphiphilic diblock copolymer polyethylene glycol-polycaprolactone (PEG-PCL) loaded with CuNC(Octa) dye for PTT and PAI. CuNC(Octa), a naphthalocyanine/phthalocyanine variant, served as the PTT agent (Figure 18).

The CuNC(Octa)-loaded micelles presented a strong PA signal and high photothermal efficiency and photostability. The micelles retained their ability to generate heat through multiple rounds of irradiation and demonstrated little ROS generation. The micelles were also stable for at least 10 days, with no signs of aggregation or precipitation. In vitro studies showed that the treatment of murine breast cancer 4T1 cells with the CuNC(Octa)-loaded micelles in combination with 808 nm laser light decreased cell viability in a dose-dependent manner. In vivo studies revealed that 24 h after the treatment of Balb/c mice bearing murine 4T1 xenografts with the micelles and irradiation with a laser light, the accumulation of the micelles in the tumors was readily detectable by PA imaging. Moreover, the CuNC(Octa)-loaded micelles demonstrated efficient heat generation ability with PTT, resulting in the complete ablation of tumors.

**Figure 19:**
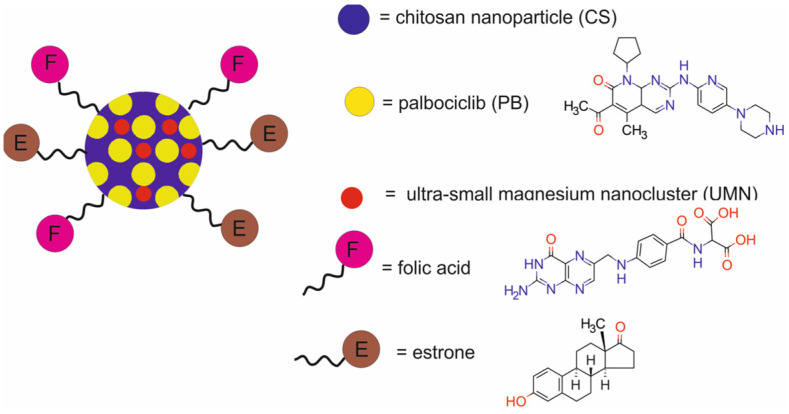
Schematic representation of the PB-UMN-CS-ES-FA-NP nanotheranostic system.

A study by Mehata et al. [62] reported PB-UMN-CS-ES-FA-NPs, a dual-receptor-targeted nanotheranostic system composed of chitosan (CS) nanoparticles functionalized with folic acid (FA) and estrone (ES) and loaded with palbociclib (PB) and ultra-small magnesium nanoclusters (UMNs) for chemotherapy and FI (Figure 19).

The physicochemical characterization of PB-UMN-CS-FA-ES-NPs demonstrated the rapid release of PB from the nano-carrier at pH 4.5. Conversely, at pH 7.4, the release of CS was less pronounced and resulted in gradual drug diffusion. In vitro studies revealed that the shape and size of blood cells were not significantly altered by exposure to PB-UMN-CS-FA-ES-NPs. The presence of the receptor-targeting group significantly elevated the toxic efficacy of PB. The exposure of MCF-7 cells to PB-UMN-CS-FA-ES-NPs induced a 54.17-fold increase in cytotoxicity, while the exposure of T-47D cells induced a 42.23-fold increase in cytotoxicity. Cellular uptake of PB-UMN-CS-FA-ES-NPs was improved in comparison to nontargeted NPs. Flow cytometry analysis demonstrated that PB-UMN-CS-FA-ES-NPs arrested cells in the G1 phase of the cell cycle. In vivo studies demonstrated tumor-specific accumulation of PB-UMN-CS-FA-ES-NPs 6 h after administration, and the nanoparticles were shown to have a remarkable capability to totally eradicate rat breast tumors during 6-day therapy. Additionally, ultrasound and photoacoustic (USG/PA) imaging demonstrated that PB-UMN-CS-FA-ES-NPs effectively reduced hypoxic tumor volume and significantly suppressed tumor vascularity as compared to nontargeted nanoparticles and free PB. Furthermore, in vitro hemocompatibility and histopathological studies confirmed the biocompatibility of the developed nanotheranostic system.

#### 4.2.2. Silica-Based Cancer Nanotheranostic Systems

Among the various types of non-metallic nanoparticles, mesoporous silica-based nanoparticles have engrossed considerable attention as a worthwhile platform for a plethora of anticancer agents, genes, proteins, radionuclides, contrast agents, or fluorescent probes due to their unique properties, such as large surface area, tunability of pore size and shape, substantial loading capacity, easy surface modification with various targeting ligands and stimuli-responsive moieties, hydrophilicity, low toxicity, and biodegradability [98,99]. Moreover, silica-based nanoparticles can incorporate different imaging and therapeutic agents into their pores or on their surface to achieve nanotheranostic capabilities [9].

**Figure 20:**
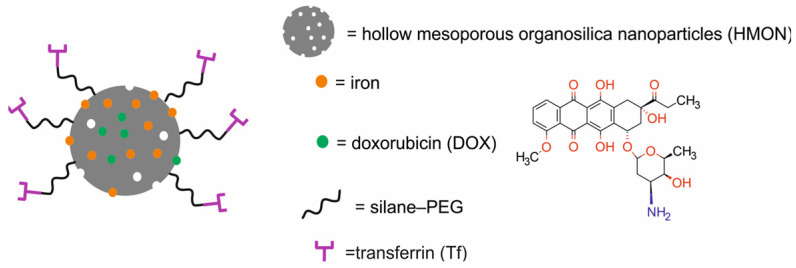
Schematic representation of the DOX@Fe-HMON-Tf nanotheranostic system.

An interesting approach to a silica-based nanotheranostic system was proposed by Zhou et al. [63]. This intelligent DOX@Fe-HMON-Tf system was composed of hollow mesoporous organosilica nanoparticles (HMONs) doped with iron (Fe), modified with silane–PEG, functionalized with transferrin (Tf), and loaded with DOX for oxygen species-mediated chemodynamic therapy (CDT) and MRI (Figure 20).

DOX served as a chemotherapeutic agent, self-generating H_2_O_2_ and eliminating GSH for efficient ferroptosis. Tf served as a targeting molecule for Tf receptors (TfRs), upregulated in many human cancer cells, facilitating delivery to tumor cells and the achievement of high intracellular DOX accumulation. Physicochemical studies demonstrated the excellent chemical stability of DOX@Fe-HMON-Tf at pH 7.4. The total DOX leakage from the nanoparticles was < 20% after 24 h incubation at pH 5.5, 6.8, and 7.4. However, incubation of the DOX@Fe-HMON-Tf NPs in 10 mM GSH solution at pH 5.5, 6.8, and 7.4 for 24 h led to ~80% release of DOX. The ICP-MS results demonstrated a slow iron release rate at all three pH values, which increased to ~75% in 10 mM GSH at all three pH values. When the culture period was prolonged to 14 days, the DOX@Fe-HMON-Tf NPs fragmented and gradually disappeared under physiological conditions. In vitro studies demonstrated a concentration-dependent cytotoxicity of the DOX@Fe-HMON-Tf NPs towards human fetal hepatocyte L-02 cells and human hepatoma cancer HepG2 cells. In vitro results also showed that the DOX@Fe-HMON-Tf NPs efficiently delivered DOX into the cells, increasing intracellular H_2_O_2_ production and diffused lipid peroxidation while presenting excellent T2-weighted MRI performance. In vivo studies in Balb/c nude mice bearing HepG2 human liver cancer xenografts demonstrated that the functionalized DOX@Fe-HMON-Tf NPs were more effectively accumulated in the tumor tissues than unfunctionalized DOX@Fe-HMON-PEG NPs. Moreover, in vivo studies demonstrated that the DOX@Fe-HMON-Tf Nps effectively inhibited tumor growth by combining ferroptosis and chemotherapy-induced cell death.

**Figure 21:**
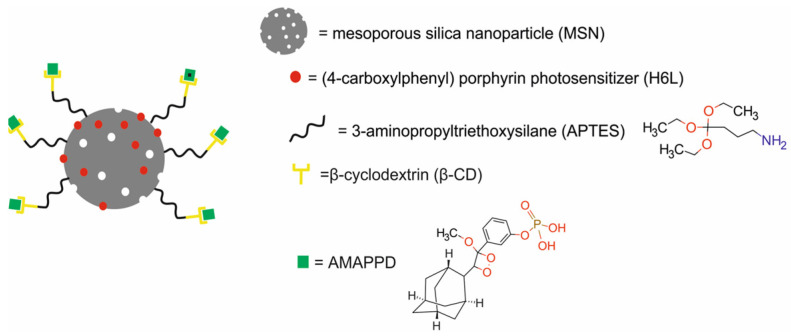
Schematic representation of MSN@H6L@β-CD@AMPPD nanotheranostics platform.

Fan et al. [64] constructed a MSN@H6L@β-CD@AMPPD nanotheranostics platform composed of mesoporous silica nanoparticles (MSNs) modified with 3-aminopropyltriethoxysilane (APTES), β-cyclodextrin (β-CD), and 3-[(2-spiroadamatane)-4-methoxy-4-(3-phosphoryloxy]-phenyl-1,2-dioxetane]dioxetane (AMAPPD) and loaded with a (4-carboxylphenyl) porphyrin (H6L) photosensitizer for chemiluminescence-mediated photodynamic therapy (CL-PDT) and near-infrared fluorescence (NIRF) (Figure 21).

In vitro results revealed that AMPPD was specifically hydrolyzed by alkaline phosphatase, highly expressed in liver cancers, which resulted in the cancer cells’ chemiluminescence. Subsequently, the H6L photosensitizer was excited by chemiluminescence through chemiluminescence resonance energy transfer (CRET), which resulted in NIRF and the generation of ^1^O_2_. CRET was confirmed by a red shift in chemiluminescence to NIRF, which is more suitable for imaging in deep tissue. The results also demonstrated that the coverage of β-CDs improved CRET’s efficiency and effectively encapsulated H6L in MSNs, preventing its leakage and avoiding damage to normal tissues. In vitro studies revealed that MSN@H6L@β-CD@AMPPD efficiently targeted and killed human hepatocarcinoma SMCC-7721 cells but not human normal liver HL-7702 cells. In vivo studies in BALB/c nude mice bearing SMCC-7721 xenografts confirmed that MSN@H6L@β-CD@AMPPD targeted tumor cells and was turned on by alkaline phosphatase, which lighted up the tumor site and enabled the imaging and diagnosis of liver cancer without any external light source. Moreover, these results proved that MSN@H6L@β-CD@AMPPD possessed an excellent PDT effectiveness in liver cancer therapy.

**Figure 22:**
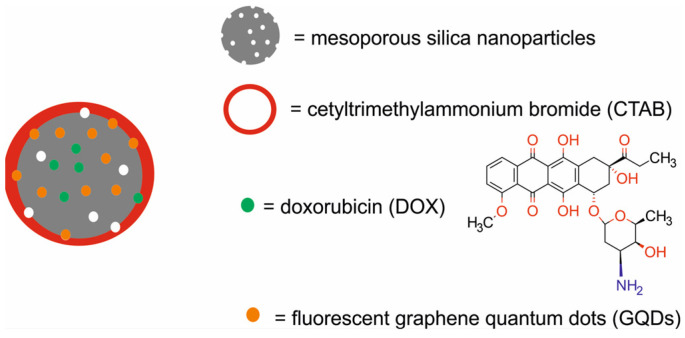
Schematic representation of DOX–carbanosilica nanotheranostic system.

In 2021, Prasad et al. [65] designed and investigated a DOX–carbanosilica nanotheranostic system composed of MSN modified with cetyltrimethylammonium bromide (CTAB), decorated with fluorescent graphene quantum dots (GQDs), and loaded with DOX for PTT, chemotherapy, and NIRF (Figure 22). Physicochemical analyses of the designed carbanosilica system demonstrated the effective distribution of GQDs in the silica frame, a high surface area (~850 m^2^/g with a pore volume of ~0.4 cm^3^/g), about 31% of drug-loading capacity, and stability. The stimuli-triggered release of DOX from carbanosilica revealed negligible DOX release (1.5%) in physiological conditions (pH 7.4 at 37 °C), whereas about 58% was observed in the case of pH 5 at 37 °C after 24 h; this was further improved (about 93%) after NIR light exposure due to the effect of the generated heat.

The photothermal performance of the DOX–carbanosilica nanohybrids showed a constant dose-dependent and laser exposure time-dependent rise in temperature. Finally, hyperthermia temperature (43 °C) was achieved in less than 2 min of NIR light irradiation. In vitro studies of carbanosilica demonstrated its successful cellular uptake and significant biocompatibility in fibroblastic L929 normal mouse adipose tissue cells. The in vitro therapeutic capacity of DOX–carbanosilica against mouse breast cancer 4T1 cells before and after NIR light exposure clearly demonstrated the potential of NIR light-triggered chemotherapy in breast cancer cells. In vivo studies demonstrated a progressive tumor regression in Balb/c mice bearing murine 4T1 xenografts after NIR light irradiation with a single dose of DOX–carbanosilica. A single-dose administration of carbanosilica demonstrated a temperature rise to about 55 °C and high fluorescent intensity, which were sufficient for tumor ablation (about 70%) and successive follow-up tumor imaging.

**Figure 23:**
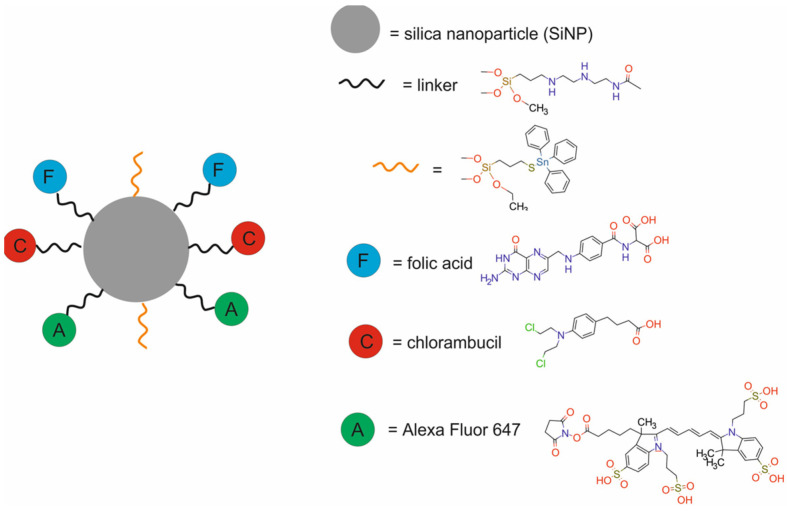
Schematic representation of the FS-DT-Chl-FA-Sn-AX nanotheranostic system.

Ovejero-Paredes et al. [66] developed a FS-DT-Chl-FA-Sn-AX nanotheranostic system composed of fibrous silica (FS) particles modified with trimethoxysilyl–propyldiethylenetriamine (DT), functionalized with two different cytotoxic agents, chlorambucil (Chl) and organotin(IV) complex (Sn), decorated with folic acid (FA), and labeled with Alexa Fluor 647 (AX) for chemotherapy and FI (Figure 23).

In vitro results revealed that the Sn-functionalized FS showed higher toxicity than silica materials bearing only Chl in triple-negative human breast cancer MDA-MB-231 cells. The wound healing assay revealed that the Sn-functionalized material showed higher antimigration properties in comparison with the Chl-functionalized nanodrug. In vivo imaging showed a selective tumor accumulation of FS-DT-Chl-FA-Sn-AX within the tumor area, the liver, and in the bowel, and to a lesser extent, in the bladder in NOD Scid IL2 receptor gamma chain KO mice bearing MDA-MB-231 xenografts. Upon FS-DT-Chl-FA-Sn-AX treatment, the tumor mass increased only 1.8-fold in comparison with mice treated with saline (3.8 fold), showing antitumor activity. In vivo biochemical studies of FS-DT-Chl-FA-Sn-AX showed no hepatic and renal damage.

**Figure 24:**
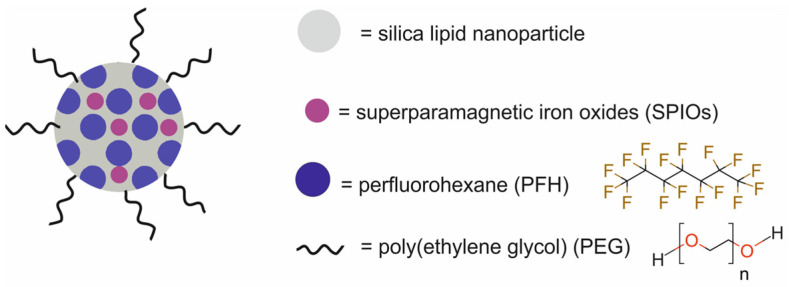
Schematic representation of the SSPN nanotheranostic system.

More recently, Liu et al. [67] prepared a multifunctional nanotheranostic system SSPN by loading superparamagnetic iron oxides (SPIOs) and perfluorohexane (PFH) in silica lipid nanoparticles modified with PEG for high-intensity focused ultrasound (HIFU) ablation of tumors, MRI, and ultrasound imaging (USI) (Figure 24). PFH served as the HIFU and as the ultrasound contrast performance. SPIOs were used as the MRI contrast agent.

Physicochemical analyses of the SSPN nanoparticles confirmed their uniform structure and excellent stability. Analysis of the SSPN response to temperature from 25 to 65 °C revealed the formation of a bubble and the transition of the PFH from the liquid phase to the gas phase, demonstrating the suitability of the SSPN for use in HIFU ablation and USI. In vitro USI results revealed that SSPNs had the potential to enhance ultrasound contrast due to the cavitation induced by bubbles. Furthermore, in vitro MRI evidently suggested that due to their SPIO content, SSPNs could be used as an excellent MRI contrast agent. In vivo studies in BALB/c mice bearing murine CT26 xenografts revealed SSPN accumulation at the tumor site due to the enhanced permeability and retention (EPR) effect. Furthermore, SSPNs could be used not only for USI and T2-weighted MRI but also as a therapeutic agent for HIFU therapy. Ex vivo HIFU experiments on degassed pig livers and in vivo HIFU experiments on BALB/c mice bearing murine CT26 xenografts confirmed that SSPNs were an effective therapeutic agent for the HIFU ablation of tumors.

#### 4.2.3. Liposome-Based Cancer Nanotheranostic Systems

Liposomes are self-assembled, (phospho)lipid-based amphipathic molecules that form unilamellar vesicles with one bilayer membrane, oligolamellar vesicles with 2–5 bilayer membranes, or multilamellar vesicles with many bilayer membranes, in which the hydrophilic head groups face the exterior aqueous environment and the hydrocarbon chains assemble within the hydrophobic interior [100]. Liposomes exhibit outstanding properties, such as high stability, high encapsulation efficiency, ability to protect the payload from physiological degradation, controlled release of drug molecules, long circulation property after the application of polyethylene glycol (PEG), composition-dependent blood clearance rate, half-life, and pharmacokinetics, and excellent biocompatibility and safety. With these advantages, liposomes have been utilized as imaging and therapeutic platforms in many studies [101]; recently, some of them included the use of liposomes for theranostic applications [102].

**Figure 25:**
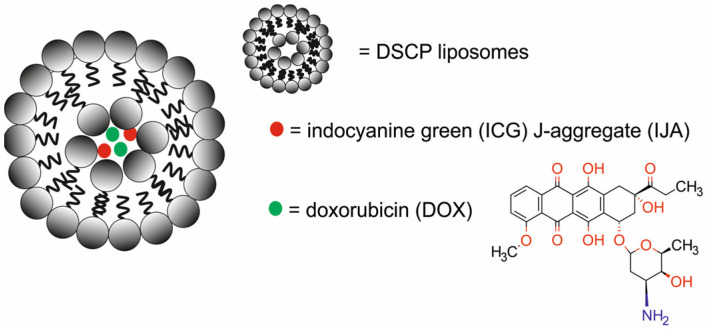
Schematic representation of the DOX-DSPC-IJA-HBS nanotheranostic system.

Cheung et al. [68] reported a simple approach to engineer a DOX-DSPC-IJA-HBS nanotheranostic system composed of DSCP liposomes (1,2-distearoyl-sn-glycero-3-phosphatidylcholine-[methoxy(polyethylene glycol)-2000) with indocyanine green (ICG) J-aggregate (IJA) incorporated in a lipid bilayer and loaded with DOX for PTT, chemotherapy, and NIRF imaging (Figure 25). The ICG served as a NIRF dye, whereas the J-aggregates protected the ICG from photo-degradation upon irradiation.

Physicochemical and spectroscopic studies of DOX-DSPC-IJA-HBS confirmed that cholesterol-containing rigid DSPC liposomes promoted IJA formation and enhanced the photothermal conversion efficiency and thermal stability of IJA, both free and in liposomal formulation. Moreover, the results demonstrated that DOX was efficiently loaded in the liposomes, with an efficiency close to that of the clinically approved Doxil^®^/Caelyx^®^ formulation. In vivo studies of the DSPC-IJA-HBS nanoparticles in BALB/c mice bearing CT 26 murine colon xenografts, BALB/c mice bearing murine breast cancer 4T1 xenografts, and NSG mice bearing C4-2B xenografts demonstrated an intermediate fluorescence at 1 h post-injection, with a constant increase in the liver, spleen, intestine, and tumor tissue up to 24 h post-injection. Fluorescence intensity was reduced at 48 h post-injection, but high intensity was still present at the tumor site, indicating high tumor retention of DSPC-IJA-HBS. Furthermore, in vivo photothermal studies demonstrated the high thermal stability of DSPC-IJA after multiple cycles of 808 nm laser irradiation.

**Figure 26:**
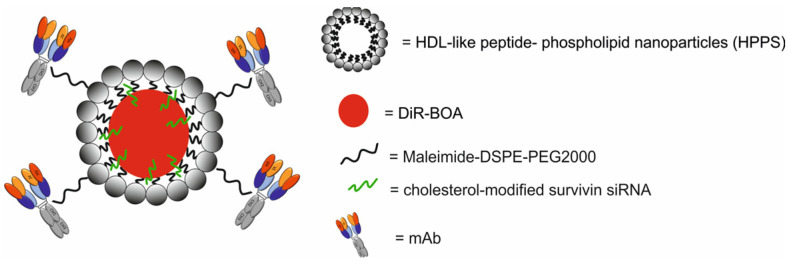
Schematic representation of the DiR-BOA-HPPS-mAb/siRNA nanotheranostic system.

A study published by He et al. [63] described a DiR-BOA-HPPS-mAb/siRNA nanotheranostic system composed of DiR-BOA (1,1′-dioctadecyl-3,3,3′,3′-tetramethylindotri-carbocyanine iodide bisoleate) loaded with HDL-like peptide–phospholipid nanoparticles (HPPS) functionalized with antibody (mAb) against TfR and loaded with cholesterol-modified survivin siRNA for therapy via the inhibition of survivin expression at the RNA level and induction of apoptosis, as well as via NIRF imaging (Figure 26).

The HPPS imitated the structural and functional properties of plasma-derived HDL. DiR-BOA, a lipid-anchored NIR fluorophore, served as a model drug cargo and diagnostic moiety for optical identification. The mAb against TfR served as a targeting ligand. In vitro studies demonstrated that the HPPS-mAb was efficiently taken up in a dose-dependent manner by various TfR-expressing tumor cell lines, including CHO cells stably expressing a hybrid of human TfR and green fluorescent protein (CHO-hTfR), human hepatic carcinoma HepG2 cells, human glioblastoma U87 cells, human cervical cancer HeLa cells, and human breast cancer MDA-MB-231 cells. The in vivo therapeutic properties of HPPS-mAb were tested in a double-tumor-engrafted mouse model with CHOvec (TfR−) and CHO-hTfR (TfR+) xenografts. The results demonstrated different distribution patterns between TfR+ and TfR− tissues. A higher accumulation of DiR-BOA-HPPS-mAb/siRNA was observed in CHO-hTfR tumors at 12 h post-systemic administration. Moreover, the results revealed that DiR-BOA-HPPS-mAb/siRNA decreased survivin expression at the RNA and protein levels in a dose-dependent manner in HepG2 cells and induced apoptosis.

**Figure 27:**
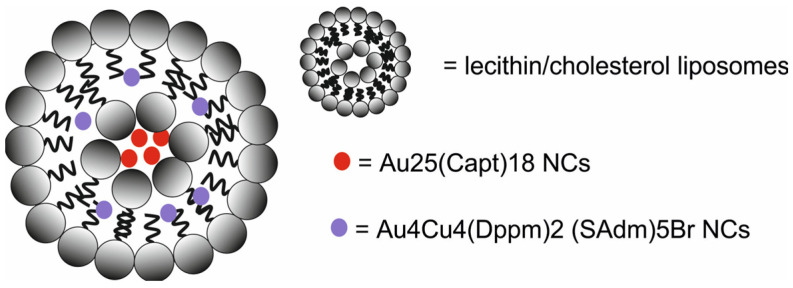
Schematic representation of the Au_4_Cu_4_/Au_25_@Lip nanotheranostic system.

Liu et al. [70] developed a Au_4_Cu_4_/Au_25_@Lip nanotheranostic system composed of lecithin/cholesterol liposomes (Lip) co-loaded with oil-soluble Au_4_Cu_4_(bis(diphenylphosphino)methane)_2_(1-adamantanethiol)_5_Br nanoclusters (Au_4_Cu_4_(Dppm)_2_(SAdm)_5_ Br NCs and water-soluble Au_25_(Capt)_18_ NCs for PTI/FI-guided PTT)/PDT (Figure 27).

The prepared Au_4_Cu_4_/Au_25_@Lip nanotheranostic system presented high stability, passive targeting due to the EPR effect, and high biocompatibility in human cervical cancer HeLa cells. Moreover, the prepared Au_4_Cu_4_/Au_25_@Lip nanotheranostic system had the ability to catalyze the decomposition of H_2_O_2_ for O_2_ production, indicating that the Au_25_ NCs contributed to the nanoplatform’s ability to perform H_2_O_2_ decomposition. In the presence of NIR laser illumination, a good photodynamic effect of Au_4_Cu_4_@Lip was observed. When H_2_O_2_ was added, the catalytic effect of Au_4_Cu_4_@Lip was increased, leading to the production of O_2_ and an enhanced photodynamic effect. In vitro experiments confirmed the production of ROS and an enhancement of the photodynamic effect by the Au_4_Cu_4_/Au_25_@ in HeLa cells. In vitro experiments revealed that the survival of HeLa cells decreased following the exposure of the cells to the Au_4_Cu_4_/Au_25_@Lip in a dose-dependent manner. The survival of cells decreased in cells treated with H_2_O_2_ after laser irradiation because of enhanced O_2_ generation. In vitro imaging results demonstrated that Au_4_Cu_4_/Au_25_@Lip was well distributed in HeLa cells, which proved its outstanding FI ability. The in vivo FI capability of Au_4_Cu_4_/Au_25_@Lip in Kunming mice bearing hepatocellular carcinoma H22 xenografts confirmed that the Au_4_Cu_4_/Au_25_@Lip allowed for tumor diagnosis by FI. Furthermore, in vivo studies demonstrated that the Au_4_Cu_4_/Au_25_@Lip possessed not only excellent real-time PTI performance for tumor diagnosis but also a great photothermal effect for tumor therapy. In addition, in vivo studies revealed that Au_4_Cu_4_/Au_25_@Lip under laser irradiation possessed an excellent PDT/PTT effect and inhibited tumor growth in mice. To sum up, Au_4_Cu_4_/Au_25_@Lip under irradiation with NIR laser exhibited three different functions: that of a photosensitizer for PDT, catalyst for O_2_ generation in the presence of endogenic H_2_O_2_, and effective agent for FI.

**Figure 28:**
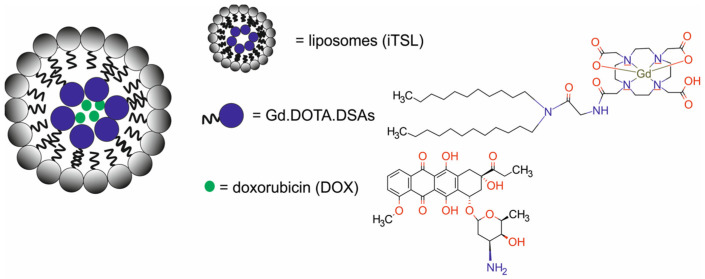
Schematic representation of the thermosensitive iTSL-DOX nanotheranostic system.

Amrahli et al. [71] presented a novel thermosensitive iTSL-DOX nanotheranostic system composed of liposomes (iTSLs) conjugated with a NIRF probe (CF750.DSA), modified with the chelator DOTA, labeled with gadolinium-based paramagnetic MRI contrast agents (Gd.DOTA.DSA), and loaded with DOX for MR-guided high-intensity focused ultrasound hyperthermia (MR-FUS), chemotherapy, and NIRF imaging (Figure 28).

The iTSL liposomes (iTSLs) were composed of a mixture of Gd.DOTA.DSA, 1,2-Dipalmitoyl-sn-glycero-3-phosphocholine (DPPC), 1,2-distearoyl-sn-glycero-3-phosphocholine (DSPC), 1-stearoyl-snglycero-3-phosphocholine (MSPC), and (ω-methoxy-polyethyleneglycol2000)-N-carboxy-1,2-distearoyl-sn-glycero-3-phosphoethanolamine (DSPE–PEG2000) at 30/54/5/5/6/0.05 mol%. Gd.DOTA.DSA served as the MR contrast agent, while CF750.DSA served as the NIFR label. Physicochemical studies revealed that DOX release was thermally induced, as indicated by a sudden increase in fluorescence intensity over 41 °C, with more than 80% of the encapsulated drug released in 2 min at 43 °C. Moreover, the DOX release profile was dependent on acoustic power, similarly to other thermal-induced release studies. In vitro MR relaxivity studies in MDA-MB-231 cells showed an enhancement of signal intensity dependent on the concentration of iTSL-DOX, but lower in terms of T1-CA compared to Gadovist^®^—one of the most efficient small-molecule cyclic gadolinium CA used in clinic. Pharmacokinetic studies of iTSL-DOX in CD-1 mice demonstrated slow DOX leakage under blood-like conditions. T1-weighted MRI of iTSLs in athymic nude mice bearing MDA-MB-231 xenografts revealed increased MR signal intensity in the tumor within an hour. Moreover, the exposure of cells to iTSL-DOX induced rapid FUS-induced drug release that was dependent on the acoustic power applied. In addition, FUS significantly enhanced iTSL-DOX uptake to the tumor and triggered a near-total release of encapsulated DOX. A significant growth inhibition of tumors was observed in the TNBC mouse model. The results also revealed that tracking iTSLs in tumors using MRI assisted in the application of FUS for precise drug release and therapy.

#### 4.2.4. Dendrimer-Based Cancer Nanotheranostic Systems

Dendrimers are macromolecular 3D-shaped structures made of a polymeric material possessing three very well-defined structural regions: a central core, branches with multiple internal repeating units covalently bonded to the core (generations), and terminal functional groups present on the surface [103]. The dendrimer core can be used for the encapsulation of drugs, bioactive agents, and contrast and/or imaging agents, whereas the terminal functional groups can be used for modification [104]. Due to these exceptional structural advantages, dendrimers have a high capability for the entrapping and/or conjugation of hydrophilic/hydrophobic entities, high loading capacity, receptor-mediated targeting, controlled drug release, and good biodistribution [105]. Owing to these properties, dendrimers have found many applications in medicine, e.g., as drug delivery systems, gene transfection enhancers, contrast/imaging agents, and nanotheranostics [106].

**Figure 29:**
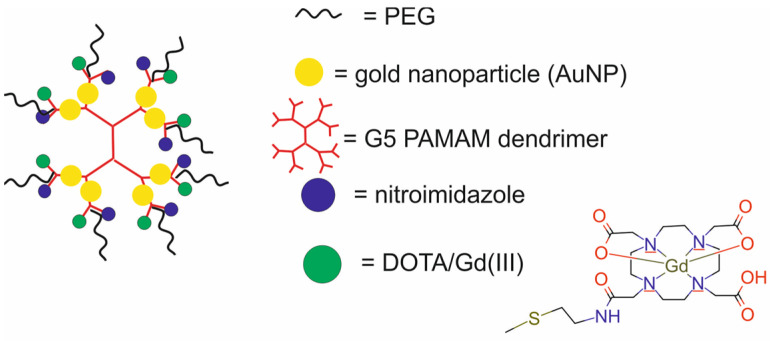
Schematic representation of the Gd-Au-DENPs-Nit nanotheranostic system.

In the quest for new nanotheranostic systems using dendrimers, Fan et al. [72] reported the creation of a hypoxia-targeted Gd-Au-DENPs-Nit nanotheranostic system, composed of G5 PAMAM dendrimers with entrapped AuNPs, linked with 2,2′,2″-(10-(2-(2,5-dioxopyrrolidin-1-yloxy)-2-oxoethyl)-1,4,7,10-tetraazacyclododecane-1,4,7-triyl) triacetic acid (DOTA-NHS), functionalized with nitroimidazole via a PEG linker, and labeled with Gd(III) by a DOTA chelator for CT/MR dual-mode imaging and enhanced radiotherapy of hypoxic tumors (Figure 29). Nitroimidazole served as a hypoxia-targeting agent. AuNPs served as a CT imaging agent, whereas Gd(III) served as an MRI agent.

In vitro studies demonstrated a very good biocompatibility of the Gd-Au DENPs-Nit, with over 80% viability of nasopharyngeal carcinoma CNE-1 cells, nasopharyngeal carcinoma hypoxia-resistant CNE-1H cells, and normal NIH3T3 cells. Moreover, CNE-1H cells incubated with Gd-Au DENPs-Nit exhibited a much higher Au content than those incubated with Nit-free Gd-Au DENPs. The efficient endocytosis of Gd-Au DENPs-Nit into CNE-1H cells was confirmed by bio-TEM images. In vivo CT/MR dual-mode imaging and quantitative tumor CT revealed an excellent X-ray attenuation effect and acceptable r1 relaxivity in BALB/c nude mice bearing hypoxia-resistant CNE-1H xenografts, confirming that Gd-Au DENPs-Nit enabled targeted dual-mode CT/MR imaging. Biodistribution studies of the Gd-Au DENPs-Nit demonstrated the accumulation of Au in the heart, liver, spleen, and lung, accompanied by a relatively small amount of Au in the tumor tissue. Au accumulation in the tumor tissue of mice treated with Gd-Au-DENPs-Nit was much higher than that in mice treated with Gd-Au-DENPs, suggesting a good hypoxia-targeting ability. Furthermore, in vitro and in vivo tumor hypoxia studies revealed that Gd-Au-DENPs-Nit was an effective sensitizer to reinforce the radiotherapy response of hypoxic tumors via an enhancement of the intracellular generation of ROS, DNA damage, and prevention of DNA repair, which resulted in a smaller tumor volume than in the radiotherapy-alone group.

**Figure 30:**
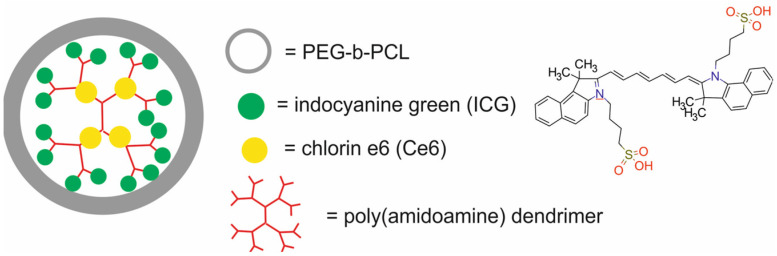
Schematic representation of the SNP_ICG/Ce6_ nanoplatform.

Wang et al. [73] constructed a light-triggered size-switchable PEG-b-PCL-TK-PAMAM-ICG-Ce6 (SNP_ICG/Ce6_) nanoplatform composed of poly(amidoamine) dendrimers with indocyanine green (PAMAM-ICG) conjugated to polymer poly(ethylene glycol)-b-poly(ε-caprolac-tone) (PEG-b-PCL) through a thioketal (TK) bond and loaded with Ce6 for PTT, PDT, and FI. The ICG served as a photothermal agent in PTT, whereas the Ce6 was used as a photosensitizer in PDT (Figure 30).

Physicochemical analyses confirmed the successful loading of Ce6 and ICG into the SNP_ICG/Ce6_, which showed good stability, dispersibility, ^1^O_2_ generation capacity, and photothermal properties. Moreover, the results showed that ^1^O_2_ generated by the encapsulated Ce6 upon laser irradiation cleaved the TK bond rapidly, leading to a size change from 118 nm to 10 nm in the SNP_ICG/Ce6_. In vitro confocal imaging and fluorescence intensity analysis revealed that the SNP_ICG/Ce6_ was efficiently internalized by murine sarcoma 4T1 cells. Moreover, the results demonstrated that the SNP_ICG/Ce6_ generated ^1^O_2_ under normoxic conditions upon laser irradiation, but the generation of ^1^O_2_ was strongly suppressed under hypoxic conditions in 4T1 cells. The results also indicated that PTT effectively killed 4T1 cells under normoxic and hypoxic conditions, but PDT killed 4T1 cells effectively only under normoxic conditions. Biodistribution studies in BALB/c nude mice bearing 4T1 xenografts revealed that SNP_ICG/Ce6_ accumulated at the perivascular sites of the tumor after intravenous injection and remained there for up to 36 h. Finally, the site-specific combination of PDT and PTT therapies in mice bearing 4T1 xenografts showed efficient tumor inhibition in a hypoxic microenvironment (~80%) at a mild temperature after irradiation with an 808 nm laser, with no obvious systemic side effects.

**Figure 31:**
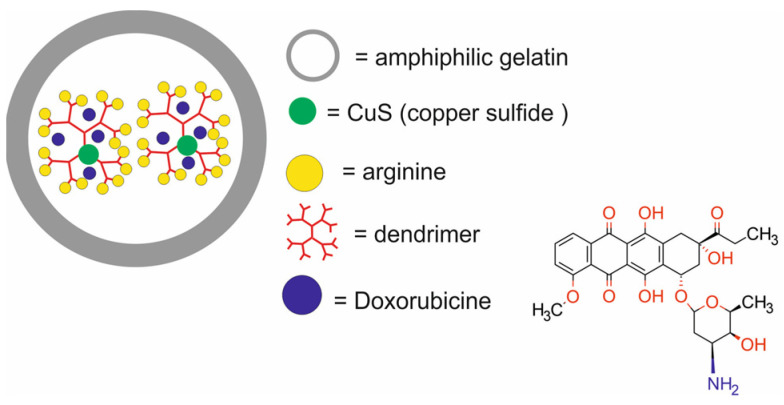
Schematic representation of the PRDCuS@AG nanotheranostic system.

Pan et al. [74] developed a biodegradable PRDCuS@AG nanotheranostic system composed of arginine-conjugated G4 PAMAM dendrimers (PR) wrapped by amphiphilic gelatin (AG) and loaded with DOX and copper sulfide (CuS) for deep tumor chemo-/phototherapy guided by photoacoustic imaging (PI) (Figure 31). The copper sulfide (CuS) served as a photoacoustic (PA) agent, whereas arginine was used to improve the penetration of PRDCuS@AG through the membrane of cancer cells.

Experiments demonstrated that PRDCuS@AG had strong absorption in the NIR region, indicating that it can efficiently convert NIR light energy into heat. Moreover, PRDCuS@AG underwent rapid biodegradation into 3 nm ultra-small PRDCuS nanoparticles due to the acceleration of matrix metalloprotease 9 (MMP9) activity by NIR light, thus achieving an effective penetration of multicellular tumor spheres. In vitro studies proved the excellent cell-killing ability of PRDCuS@AG under NIR light irradiation due to effective synergistic chemo-/phototherapy in 4T1 cells, compared with PRDCuS@AG without NIR or with DOX-free PRCuS irradiated with NIR. Moreover, the results demonstrated that under NIR light irradiation, PRDCuS@AG generated ROS at the cellular level, indicating that PRDCuS@AG had photodynamic properties. Photoacoustic imaging and biodistribution studies showed that in BALB/c nude mice bearing 4T1 xenografts, the accumulation of PRDCuS@AG in the tumor constantly increased and reached the maximum at 12 h after treatment. A tumor inhibition rate reaching 97% and an almost total elimination of tumors were observed 15 days after treatment. In addition, no inflammation or significant damage was observed in other major organs.

**Figure 32:**
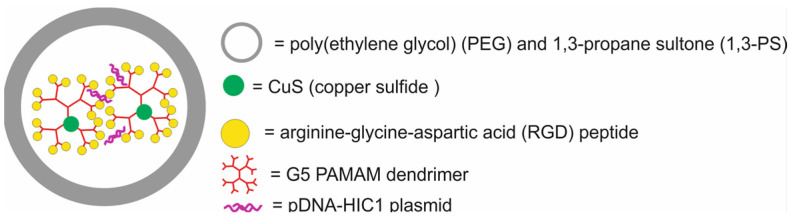
Schematic representation of the RGD-CuS DENPs/pDNA polyplex nanosystem.

Ouyang et al. [75] created an RGD-CuS DENPs/pDNA polyplex nanosystem composed of G5 PAMAM dendrimers modified with PEG and 1,3-propane sultone (1,3-PS), functionalized with arginine–glycine–aspartic acid (RGD) peptide with entrapped CuS nanoparticles (CuS DENPs) and complexed with plasmid DNA encoding hypermethylation in cancer 1 (pDNA-HIC1) for PAI-directed PPT and gene therapy (Figure 32). The copper sulfide (CuS) served as a PA agent, whereas the RGD peptide served as a targeting ligand to carry the RGD-CuS DENPs/pDNA polyplexes to the integrin αvβ3 overexpressing tumor cells. The pDNA-HIC1 plasmid was used to prevent cancer cell invasion and metastasis, the dendrimers were used as nonviral vectors, whereas the 1, 3-PS monomer was used to make the dendrimers antifouling.

Protein resistance assays confirmed that partial 1,3-PS modification rendered RGD-CuS DENPs with good protein resistance properties. The RGD-CuS DENPs exhibited high photothermal conversion efficiency and caused a continuous temperature increase upon laser irradiation, indicating their good photothermal stability. Moreover, the polyplexes had good thermal and PAI properties. In vitro uptake studies demonstrated that the RGD-modified CuS DENPs were specifically uptaken by MDA-MB-231 cells expressing αvβ3 integrin. Furthermore, the RGD-CuS DENPs/pDNA polyplexes presented enhanced anticancer activity, combining PTT and the pDNA delivery-rendered inhibition of metastasis of MDA-MB-231 cells. PA/thermal images of tumors in BALB/c-nude mice bearing MDA-MB-231 xenografts proved the RGD-mediated tumor targeting with a significant temperature increase during laser irradiation. In vivo results from combined PTT/gene therapy after the intratumoral injection of RGD-CuS DENPs and laser irradiation demonstrated an effective inhibition of primary tumors and lung metastases without tumor relapse at 14 days post-treatment. These results confirmed that the combined effect of PTT/gene therapy was superior to single-mode PTT or gene therapy treatment. Further survival rate analysis revealed that the tumor-bearing mice treated with RGD-CuS DENPs + NIR were all alive after 35 days. Finally, ex vivo tumor fluorescence images and tumor cell staining demonstrated that the polyplexes were taken up by the tumor cells quickly and efficiently.

#### 4.2.5. Carbon-Based Cancer Nanotheranostic Systems

Carbon-based nanomaterials include graphene and its derivatives, carbon quantum dots, nanodiamonds, carbon nanotubes, fullerenes, and MXenes [107]. These nanomaterials have different properties, even if they have the same composition.

Graphene and graphene-based nanomaterials consist of a monolayer of carbon atoms that comprises a honeycomb-shaped lattice [108]. They exhibit one atom thickness, equal to 0.335 nm, a large surface area, and a high aspect ratio for modifications and functionalization with several bioactive molecules. In addition, these nanomaterials possess remarkable stability, elasticity, and drug-loading capacity, significantly higher than those of other drug delivery systems. Moreover, these nanomaterials show high biocompatibility and biodegradability. Because of their unique structure and fascinating properties, graphene and graphene-based nanomaterials have gained considerable interest in studies on developing theranostic nanosystems [109]. Carbon quantum dots (CQDs) are small carbon nanoparticles with sizes less than 10 nm. Their tunable photoluminescence, chemical luminescence, fluorescence characteristics in the range from UV to NIR regions, small size, biocompatibility, and low toxicity make them an excellent candidate for theranostic applications. Nanodiamonds are carbon allotropes ranging in size from 2 to 8 nanometers, with truncated octahedrons. They exhibit a number of unique properties, including exceptional biocompatibility, small size, specific surface area with high adsorption capacity and versatile functionalization capability, high chemical stability, high photostability, and broad fluorescence emission spectrum, which make them promising for the development of cancer nanotheranostics [110]. Carbon nanotubes contain one or several layers of graphene rolled up into cylindrical tubes with a typical diameter of about 0.4 to 3 nm and a length of about 2 nm to micrometers. They have attracted an incredible interest in the biomedical field due to their high drug-loading capacity, flexible interaction with cargo, ability to release therapeutic agents at targeted sites, high surface area, considerable strength, outstanding optical and electrical features, and high stability [111]. These properties make them ideal candidates for the encapsulation and delivery of drugs, genes, and imaging agents, but their toxicity and low biodegradability are limiting factors of their use in biomedical applications [112]. Fullerenes are closed cage-like structures which possess a high surface area and versatile functionalization capability, a high ability to encapsulate and deliver cargo molecules, photoelectrochemical properties, high resistance to photobleaching, and the ability to generate highly reactive singlet oxygen or superoxide radicals by exposure to UV or visible light. However, their highly hydrophobic nature and tendency to aggregate are factors limiting their use and their full biomedical potential. MXenes are a new class of carbon-based two-dimensional materials composed of transition metal carbides, carbonitrides, and nitrides. They consist of a single layer or a few layers of graphene, graphene derivatives, black phosphorus, or graphitic carbon nitride. Their thickness is usually less than 1 nm, whereas their lateral size ranges from nanometers to micrometers. MXenes possess good biocompatibility, low toxicity, and degradability. Furthermore, MXenes have unique optical, thermal, and magnetic properties, high drug-loading capacity, and photothermal conversion capability [113]. To sum up, these unique properties of carbon-based nanomaterials make them a promising candidate for biomedical applications in drug/gene delivery, PAI, NIRI, FI, MRI, and PTT. Moreover, the ability of carbon-based nanomaterials to act as sensitizers for the photoproduction of ^1^O_2_ allows for their potential use in PDT [107].

**Figure 33:**
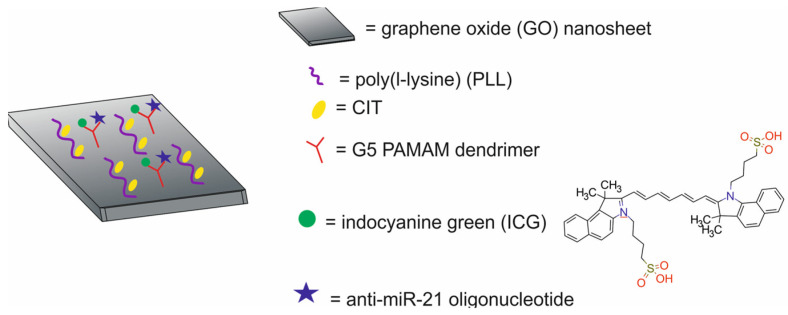
Schematic representation of the GPCP/miR-21i/ICG nanocomplex.

Thus, Wu et al. [76] developed an acid-triggered GPCP/miR-21i/ICG all-in-one nanocomplex composed of graphene oxide (GO) nanosheets modified with multilayer polymers poly(l-lysine) (PLL) conjugated with citraconic (Cit) anhydride and G4-PAMAM dendrimers (GPCP) loaded with anti-miR-21 oligonucleotide (miR-21i) and indocyanine green (ICG) for PTT, gene therapy, and PTI (Figure 33). The ICG served as a NIR fluorescent dye. The anti-miR-21 oligonucleotide was used to target miR-21 pathways.

Physicochemical studies showed that GPCP/miR-21i/ICG exhibited excellent pH-sensitive charge conversion properties. GPCP/miR-21i/ICG demonstrated a negative charge in neutral buffer, which was converted to a positive charge at pH 5.0 because of the acid sensitivity of Cit. Moreover, GPCP/miR-21i/ICG showed excellent photothermal/photochemical properties. In vitro studies demonstrated that G4-PAMAM dendrimers effectively delivered miR-21i into MDA-MB-231 cells. Fluorescence images showed that miR-21i was localized in the periplasmic regions at an early time point (4 h) and was subsequently accumulated in the cytoplasm (8 h). After 24 h of incubation, GPCP/Cy5-miR-21i was observed in endosomes/lysosomes. In vitro toxicity results demonstrated that GPCP/miR-21i/ICG in the dark slightly decreased cell viability after 24 h of treatment. However, upon laser irradiation, GPCP/miR-21i/ICG exhibited a dose-dependent inhibition of MDA-MB-231 cells. Moreover, such treatment successfully silenced endogenous miR-21, leading to a diminished level of anti-apoptotic Bcl-2 protein and upregulated levels of PTEN and Bax. In vivo biodistribution and trafficking studies in BALB/c-nude mice bearing MDA-MB-231 xenografts revealed that GPCP/miR-21i/ICG accumulated mostly in the tumors, where it was retained for a long duration. However, ex vivo fluorescence images demonstrated a strong accumulation of GPCP/miR-21i/ICG in the liver and kidney, with little or no accumulation in other healthy tissues. Furthermore, GPCP/miR-21i/ICG markedly inhibited tumor growth, with minimal toxic effects in normal tissues under NIR laser irradiation.

**Figure 34:**
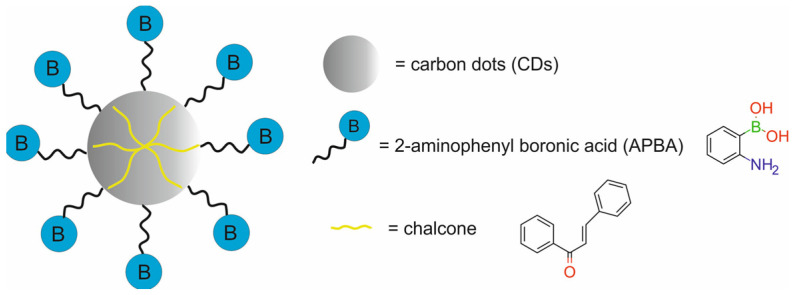
Schematic representation of the chalcone–APBA-CD nanotheranostic system.

Fahmi et al. [77] reported a new design of a chalcone–APBA-CD nanotheranostic system composed of carbon dots (CDs) labeled with fluorescent 2-aminophenyl boronic acid (APBA) and modified with chalcone for chemotherapy and FI (Figure 34). The APBA was used to accelerate photoluminescence and cancer cell-specific targeting to sialic acid on cell membrane, whereas the chalcone served as a chemotherapeutic agent and effectively halted tumor development by the inhibition of cell growth and proliferation.

Physicochemical studies of chalcone–APBA-CD proved its chemical structure, stability, excitation-dependent photoluminescence, high chalcone loading, and pH-dependent release. The loading amount and loading efficiency reached ~70 and ~100, respectively. However, the highest release of chalcone was observed in an acidic medium (pH = 4). In vitro results demonstrated that chalcone–APBA-CDs effectively decreased the viability of HeLa cells (cytotoxicity concentration, CC_50_ = 5.4 μg/mL). Moreover, flow cytometry results and confocal fluorescent images proved the effective internalization of the chalcone–APBA-CDs CDs into the cytoplasm of HeLa cells. In vivo studies revealed a remarkable reduction in tumor size in fibrosarcoma cancer-bearing mice, with no damaging effect on the other organs.

**Figure 35:**
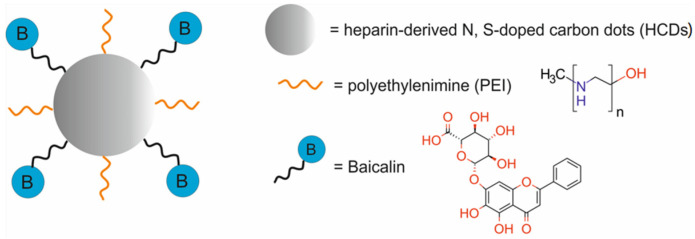
Schematic representation of the BA-PHCDs nanotheranostic system.

In a study by Meher et al. [78], biocompatible heparin-derived N,S-doped carbon dots (HCDs) modified with polyethylenimine (PEI) and loaded with baicalin (BA) [BA-PHCDs] were synthesized for chemotherapy and FI (Figure 35). Baicalin (BA), a naturally occurring compound, served as the chemotherapeutic drug. Nitrogen (N) and sulfur (S) doping on CDs was applied to enhance fluorescence efficiency. The PEI polymer was used as a surface passivating agent; it facilitated the loading of BA and also played a crucial role in the pH-responsive drug delivery.

Physicochemical analysis revealed a sustained release of BA from the BA-PHCDs (up to 80%) in a mildly acidic pH (5.5 and 6.5). However, an insignificant amount of BA was released in pH 7.4 and 8.4. In vitro studies in human lung cancer A549 cells and normal murine fibroblast NIH/3T3 cells revealed that BA-PHCDs’ toxicity could be attributed to the cytotoxic effect of BA. Moreover, cancer cells were more susceptible to BA-PHCDs than normal cells. Flow cytometry analysis demonstrated cell cycle arrest in the G2/M phase and a higher potential for apoptosis due to the increased expression level of the proapoptotic BAX protein. A bioimaging efficiency and localization study of the BA-PHCDs revealed the excellent cell penetration efficiency of PHCDs with complete cytoplasmic localization.

### 4.3. Core–Shell Nanoparticles and Nanocomposite Hybrids

Nanocomposite hybrids are heterogeneous materials in which one phase has nanoscale morphology. The phases of nanocomposites may be inorganic–inorganic, inorganic–organic, or organic–organic. They are mainly multiple nanomaterials or nanomaterials incorporated into another bulk material. The properties of nanocomposite hybrids are highly influenced by the structure, composition, interfacial interactions, and individual properties of components, which allows for the exploitation of a synergistic effect of the new nanomaterial. Among the wide variety of nanocomposite hybrids reported in the literature, metal-containing nanocomposites have garnered significant attention for theranostic applications [114,115].

**Figure 36:**
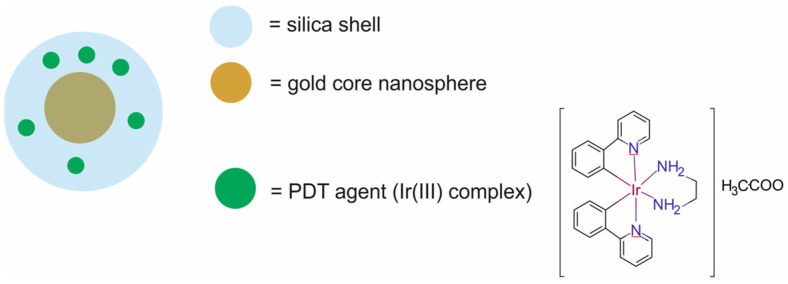
Schematic representation of the Ir1-AuSiO2 nanotheranostic system.

Ricciardi et al. [79] reported preliminary in vivo studies on an Ir_1_-AuSiO_2_ nanotheranostic system composed of core–shell silica–gold nanoparticles (AuSiO_2_) loaded with Ir_1_ for PTT and PDT imaging (LI) (Figure 36). Ir_1,_ a highly luminescent water-soluble Ir(III) complex, served as a new-generation PDT agent. The gold core was used as a light-to-heat conversion agent for PDT. The silica shell was used to confer stability to the gold nucleus and to provide a reservoir for the encapsulation of Ir_1._

Physicochemical studies demonstrated that the Ir_1_-AuSiO_2_ nanoparticles were water-soluble and exhibited long-term colloidal stability even after 18 months. The extinction spectrum of the Ir_1_-AuSiO_2_ revealed a plasmon resonant frequency at 520 nm, whereas excitation and emission spectra confirmed the Ir_1_ loading into the polysiloxane matrix. Singlet oxygen studies proved the generation of ^1^O_2_ by the Ir_1_-AuSiO_2._ The Ir_1_-AuSiO_2_ exhibited higher ^1^O_2_ generation than the silicon phthalocyanine 4 (Pc4), which is one of the most efficient phthalocyanine-based photosensitizers. In vivo studies demonstrated that the luminescent signal of the Ir_1_-AuSiO_2_ after injection into the tumors of a human glioblastoma Gli36D5 xenograft bearing-mouse model increased gradually up to 90 min and then remaining constant even at 24 h post-injection. In vivo PTT results revealed that the injection of Ir_1_-AuSiO_2_ into the tumor and irradiation at 365 nm for 15 min induced a gradual decrease in tumor size. Complete tumor regression was observed 24–26 days post-treatment without recurrence for over 100 days. Moreover, X-ray Phase Contrast Tomography investigations of ex vivo samples of Gli36D5 tumors showed clusters of Ir_1_-AuSiO_2_ nanoparticles, identified mostly in the proximity of microvessels, and highlighted a vast devascularization process by micro-vessel disruption.

**Figure 37:**
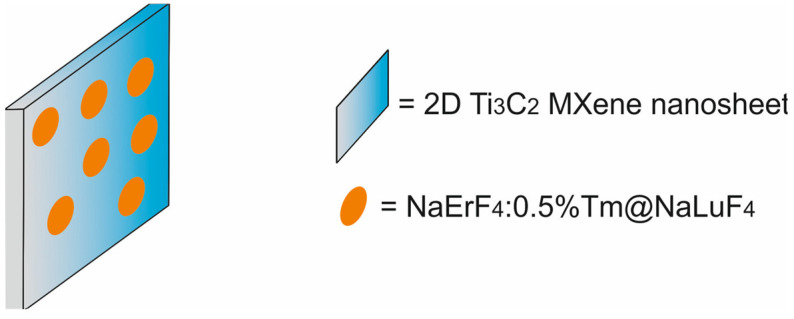
Schematic representation of the NaErF4@ Ti3C2 nanotheranostic system.

Pan et al. [80] developed a NaErF_4_@ Ti_3_C_2_ nanotheranostic system composed of 2D titanium carbide (Ti_3_C_2_) MXene nanosheets functionalized with core–shell NaErF_4_:0.5%Tm@NaLuF_4_ (NaErF_4_) nanoparticles for PTT, MRI, and FI (Figure 37). The Ti_3_C_2_ MXenes exhibit a large surface area and strong absorbance in the NIR region. The NaErF4:0.5%Tm@NaLuF4 is a new self-sensitized rare-earth nanomaterial, where Er^3+^ ions operate as both a sensitizer and activator, avoiding the concentration-quenching effect. Moreover, heavily doped Er^3+^ ions possess a large intrinsic magnetic moment. These benefits allowed NIR-IIb and MR imaging to be performed.

In vitro photothermal studies of the NaErF_4_@ Ti_3_C_2_ after exposure to 808 nm laser light demonstrated superior photothermal conversion properties and photostability. MRI and NIR-IIb imaging in vivo revealed that the pattern representing MR signal strength darkened as the concentration of the NaErF_4_@ Ti_3_C_2_ nanocomposites increased, indicating that the NaErF_4_@ Ti_3_C_2_ nanocomposite could potentially be a good contrast agent for high-field T2-weighted MRI. NIR-IIb imaging demonstrated a large tissue penetration depth and high imaging clarity of the designed nanocomposite in BALB/c-nu mice bearing HepG2 xenografts. In vivo biodistribution studies revealed an accumulation of the NaErF_4_@ Ti_3_C_2_ nanocomposite at the tumor site during whole imaging period, while the signals in the liver and spleen diminished with time. Furthermore, in vivo experiments demonstrated that, after exposure to an 808 nm laser, the NaErF_4_@ Ti_3_C_2_ nanocomposite induced significant tumor ablation without side effects on other organs.

**Figure 38:**
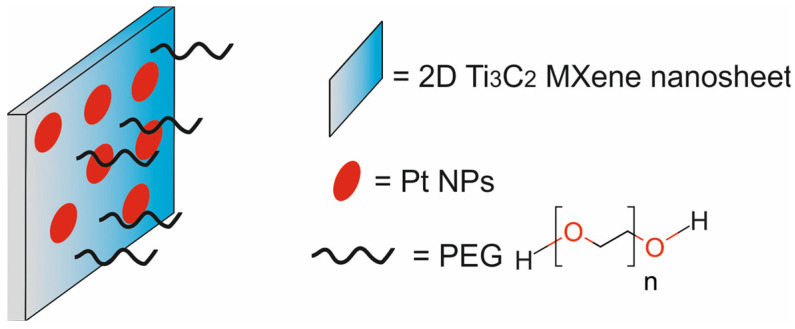
Schematic representation of the Ti_3_C_2_ Tx-Pt-PEG 2D nanocomposite.

MXene nanosheets were used also by Zhu et al. [81], who developed a Ti_3_C_2_ Tx-Pt-PEG 2D nanocomposite composed of a 2D titanium carbide (Ti_3_C_2_) MXene nanosheet modified with PEG and decorated with platinum (Pt) nanoparticles for PTT and PI (Figure 38). The Ti_3_C_2_ nanosheet was used as a substrate to anchor functional components and Pt nanoparticles and served as a PTT agent due to the improved photothermal conversion efficiency. The Pt nanoparticles served as a chemotherapy agent and artificial nanozyme with POD-like catalytic activity, leading to the decomposition of the endogenous H_2_O_2_ and generation of hydroxyl radicals (•OH).

Physicochemical analysis confirmed the successful fabrication of the Ti_3_C_2_Tx-Pt-PEG nanocomposite and demonstrated its exceptional ability to convert NIR-II light energy into thermal energy, making the nanocomposite a stable and durable photothermal agent for PTT. In addition, Ti_3_C_2_Tx-Pt-PEG at higher concentrations possessed POD-like activity, which was enhanced by photothermal treatment. The nanocomposite persistently catalyzed the decomposition of H_2_O_2_ and generated •OH. In vitro studies indicated that the Ti_3_C_2_Tx-Pt-PEG was efficiently taken up and accumulated by 4T1 cells and L929 cells. The toxicity study revealed that the Ti_3_C_2_Tx-Pt-PEG had good biocompatibility in L929 cells, but in 4T1 cells, the cytotoxicity increased with higher concentrations of the nanocomposite. Confocal images revealed that the incubation of 4T1 cells with Ti3C2Tx-Pt-PEG irradiation with 1064 nm light lead to the ablation of the 4T1 cells due to synergistic photothermal and chemodynamic effects. An in vitro experiment also confirmed that the Ti_3_C_2_Tx-Pt-PEG nanocomposites displayed a high PA signal intensity. In vivo studies in BALB/c mice bearing 4T1 xenografts demonstrated that the Ti_3_C_2_Tx-Pt-PEG under 1064 nm irradiation gradually increased the PA signal intensity of the tumor site, which attained a maximum at 6 h but was still observed even 24 h post-injection. Tumor growth in mice injected with the Ti_3_C_2_Tx-Pt-PEG nanocomposite and irradiated with 1064 nm light was significantly suppressed. The in vivo biodistribution studies of Ti_3_C_2_Tx-Pt-PEG showed high accumulation at tumor sites, in the spleen, in the liver, and in the kidneys.

**Figure 39:**
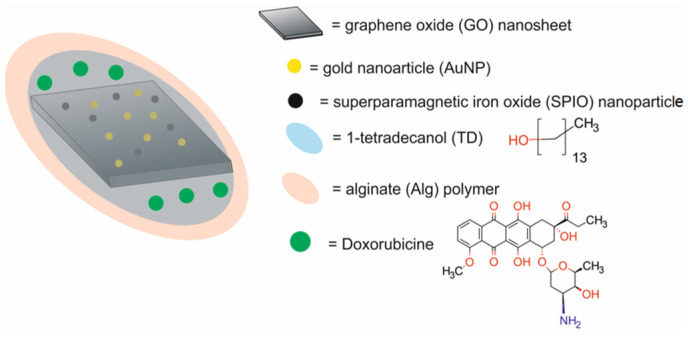
Schematic representation of the GO-SPIO-Au-DOX-TD-Alg nanotheranostic system.

More recently, Mirrahimi et al. [82] synthesized GO-SPIO-Au-DOX-TD-Alg NCs, a multifunctional stimuli-responsive smart nanocomposite composed of GO nanosheets decorated with AuNPs and SPIO nanoparticles, coated with DOX-loaded 1-tetradecanol (TD), and further modified with an alginate (Alg) polymer for PTT, chemotherapy, CT, and MR imaging (Figure 39).

The GO nanosheets served as a PTT agent and activator of phase change to trigger DOX release. TD, a phase-change material (PCM), was used to confine DOX molecules to the GO surface and served as a gate-keeper to regulate DOX release from the GO-SPIO-Au-DOX-TD-Alg nanocomposite upon NIR irradiation. The AuNPs served as a CT agent, whereas the SPIO nanoparticles were used as an MRI agent. The Alg polymer hydrogel was applied to improve the nanocomposite’s biocompatibility and colloidal stability under physiological conditions. Physicochemical analysis of GO-SPIO-Au-DOX-TD-Alg indicated the successful decoration of GO nanosheets with SPIO and Au nanoparticles and confirmed the magnetization of the nanocomposite due to the presence of SPIO. Moreover, the nanocomposite showed a concentration-dependent, significantly increased heating rate under NIR irradiation. Moreover, the results demonstrated that the PCM was able to retain DOX molecules on the surface of the NCs below its melting point. In contrast, under external heating, the GO-SPIO-Au-DOX-TD-Alg exhibited a much faster DOX release rate, resulting from PCM transformation from a solid to a liquid state. CT/MR imaging studies revealed a linear increase in CT contrast with increasing Au concentration; T2-weighted MR imaging at 3T MR revealed a concentration-dependent, high hypointense contrast, with a transverse relaxivity (r_2_) of 60.15 mM^−1^ s^−1^. These results were confirmed by in vivo CT/MR imaging studies in Balb/c mice bearing murine colon adenocarcinoma CT 26 xenografts, which indicated that the GO-SPIO-Au-DOX-TD-Alg nanocomposite can be used as a dual CT/MR contrast agent. In vitro combined PTT–chemotherapy experiments demonstrated that CT26 cells treated with GO-SPIO-Au-DOX-TD-Alg and NIR irradiation showed significantly higher cytotoxicity than cells receiving PTT alone or DOX alone. These studies also demonstrated that the synergistic tumor-killing effect of PTT and chemotherapy was a result of enhanced apoptosis. These results were confirmed by in vivo antitumor therapeutic efficacy studies, which demonstrated that tumors in Balb/c mice bearing CT 26 xenografts treated with PTT/chemotherapy were completely eradicated without showing evidence of regrowth over a 90-day follow-up period. However, despite a statistically significant prolongation of the survival rate, all mice died within two months.

**Figure 40:**
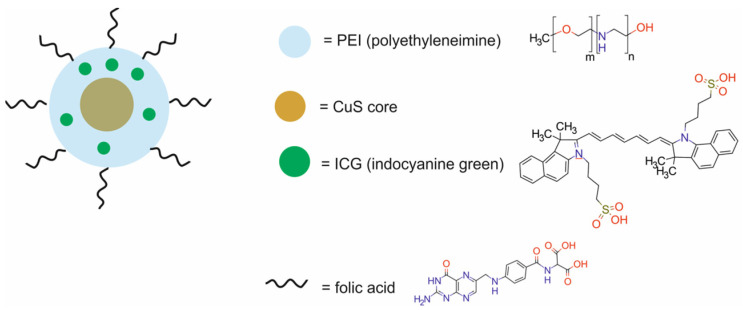
Schematic representation of the CuS-PEI-ICG-FA nanotheranostic system.

Sobhana et al. [34] formulated an ultra-small CuS-PEI-ICG-FA nanocomposite composed of copper sulfide nanoparticles (CuS NPs) coated with PEI, loaded with ICG, and functionalized with FA for PTT and PI (Figure 40). The CuS NPs served as a PTT agent. The PEI, an electrolyte cationic linear polymer, was used as a colloidal stabilizing agent with a high degree of viscidity and water solubility and facilitated the incorporation of FA on to the nanoparticle surface. The FA was used to actively target the folate receptors overexpressed on a variety of cancer cells.

Physicochemical studies confirmed an excellent binding of free ICG on the prepared CuS-PEI-ICG-FA nanocomposite, which definitely showed good photothermal properties. Moreover, these results revealed an excellent reactivity and long-term colloidal stability of CuS-PEI-ICG-FA, showing a persistent spectral range in the NIR-I window. Photothermal conversion and photostability studies of CuS-PEI-ICG-FA under exposure to 808 nm light demonstrated a significant and fast increase in temperature. The highest temperature (57 °C) was attained after 5 min. In vitro studies of the nanocomposite demonstrated a high percentage of viable HeLa cells. However, HeLa cell viability decreased in a concentration-dependent manner after treatment with the CuS-PEI-ICG-FA and exposure to 808 nm NIR light, confirming an effective photothermal effect. Moreover, the results demonstrated that this nanocomposite offered a superior acoustic contrast for PI. In vivo studies revealed a substantial tumor growth inhibition rate in Balb/c mice bearing HeLa xenografts when treated with CuS-PEI-ICG-FA and exposed to 808 nm NIR light. By the time of the follow-up period, the tumors in this PTT group were entirely eliminated with no signs of regrowth and with no significant reduction in body weight. Finally, long-term toxicity analysis of the CuS-PEI-ICG-FA nanocomposites without NIR irradiation showed no discernible organ damage or evident inflammation or necrosis in Balb/c mice bearing HeLa xenografts.

**Figure 41:**
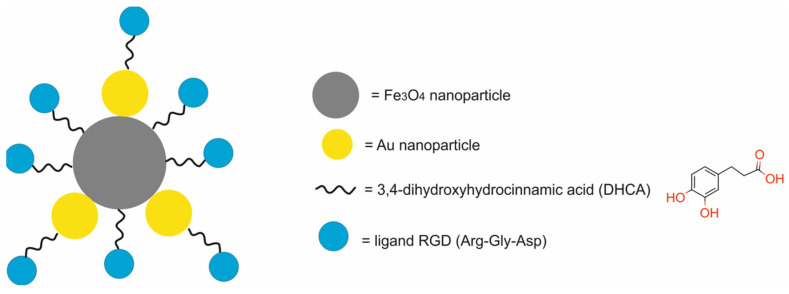
Schematic representation of the GION@RGD nanotheranostic system.

Wei et al. [83] reported the GION@RGD nanotheranostic platform, consisting of gold (Au)–iron oxide superparamagnetic (SPION) Janus nanoparticles modified with 4-dihydroxyhydrocinnamic acid (DHCA) and functionalized with the RGD ligand for PTT, therapy by NIFR-enhanced ferroptosis, and MRI (Figure 41). Superparamagnetic iron oxide nanoparticles (SIONs) served as an MRI contrast agent and additionally supplied iron ions (Fe^2+^/Fe^3+^) in the acidic tumor microenvironment and led to ferroptosis-dependent ROS generation. The Au NPs were utilized as a photothermal material to increase intracellular H_2_O_2_ levels. RGD, which is highly expressed on some tumor cells, was used as a targeting ligand that can specifically bind to the αvβ3 receptor.

In vitro studies demonstrated that the GION had good photothermal conversion capability. The temperature of the GION aqueous solution was elevated rapidly. The rise of temperature was positively correlated with the concentration of Au and laser power. Moreover, the heating performance of the GION was superior to that of AuNPs, suggesting that the immobilization of Fe_3_O_4_ nanoparticles improved the photothermal conversion efficiency of AuNPs. The results also revealed that the GION released Fe ions only under acidic conditions and participated in a Fenton reaction with H_2_O_2_ in murine sarcoma 4T1 cells. H_2_O_2_ levels in GION-treated cells significantly increased after laser irradiation and the generated H_2_O_2_ was subsequently catalyzed to •OH. In vivo biodistribution studies revealed a relatively high accumulation of GION@RGD in the liver and spleen of Balb/c nude mice bearing 4T1 xenografts. In vivo MRI studies demonstrated that the GION@RGD exhibited typical T2 contrast properties and produced an MRI signal for precise tumor detection and monitoring. The increased T2 signal was observed in the tumors of Balb/c nude mice bearing 4T1 xenografts after the iv.i. of GION@RGD. The uptake of GION@RGD was significantly higher than the uptake of nontargeted GIONs. Furthermore, in vivo results revealed that the treatment of mice with GION@RGD and laser irradiation inhibited the growth of solid tumors, with only a few side effects, confirming the synergistic treatment strategy of NIR-enhanced ferroptosis in mice.

## 5. Summary, Conclusions, and Future Perspectives

Recent progress in the field of nanotheranostic systems for cancer imaging and therapy has revealed a wide variety of design approaches that leverage different nanomaterials, different imaging and therapeutic agents, structural configurations, and functionalization strategies. These diverse designs were driven by the goal to improve cancer targeting, enhance therapeutic efficacy, overcome biological barriers, and enable multifunctionality in terms of multimode imaging and therapy. In this review paper, we summarized 40 nanotheranostic systems that were designed, synthesized, and evaluated in vitro and in vivo between 2020 and 2024.

Among these nanotheranostic systems, the most extensively explored were nanotheranostics composed of iron-based nanoparticles, gold nanoparticles, polymer nanoparticles, lipids, dendrimers, silica-based nanoparticles, carbon-based nanoparticles, and nanocomposites. Some of these nanotheranostic systems demonstrated stimuli-responsive releasing properties, including pH-responsive systems, enzyme-responsive systems, or light- and heat-responsive systems. Compared to past technologies, most of these current nanotheranostics systems enable not only conventional imaging modalities such as single-photon emission computed tomography, magnetic resonance imaging, computed tomography, or positron emission tomography but also new, upcoming imaging modalities such as photoacoustic imaging, photothermal imaging, fluorescence imaging, near-infrared imaging, or fluorescence resonance energy transfer imaging. Among the 40 discussed nanotheranostic systems, 20 possessed single-modal imaging properties, 13 possessed dual-modal imaging properties, and 2 possessed tri-modal imaging properties. Moreover, these nanotheranostic systems enabled a plethora of approaches for cancer treatment, including nanocarrier-based chemotherapy, photodynamic therapy, photothermal therapy, boron neutron capture therapy, gene therapy, high-intensity focused ultrasound therapy, enzyme therapy (peroxidase-like), therapy by inhibition of the phosphoinositide 3-kinase (PI3K/mTOR) signaling pathway, therapy by inhibition of protein expression at the RNA level, and therapy by NIFR-enhanced ferroptosis. Although many of these new nanotheranostic platforms still rely on conventional therapeutic agents, such as doxorubicin, cisplatin, or paclitaxel, some of them are loaded with innovative and promising anticancer agents, like palbocicli (a cyclin-dependent protein kinase inhibitor), chalcone (an NF-κB pathway inhibitor), baicalin (an NF-κB/STAT3 pathway inhibitor), or curcumin (a modulator of numerous cell signaling pathways). Some of them contain DNA as a therapeutic agent (e.g., anti-miR-21 oligonucleotide or plasmid DNA-encoding pDNA-HIC1) and others. Furthermore, compared to the classic approaches to cancer treatment in nanotheranostic systems, which were limited to a single therapeutic mode (e.g., chemotherapy or photothermal therapy or radiotherapy), the latest nanotheranostic systems (13 of 40) integrate multiple therapeutic modes (e.g., photothermal therapy and enzyme therapy, photothermal therapy and chemotherapy, photothermal therapy and photodynamic therapy) within a single platform. The next important advantages of these new nanotheranostic systems are their high stability and high loading efficiency, up to 90% in some nanosystems.

Despite these significant advantages, nanotheranostic systems face several limitations that may hinder their clinical translation and large-scale production. First of all, the vast majority of these nanotheranostic systems are in the premature stages of preclinical in vitro and in vivo studies, which included only a few cancer cell lines and/or a small number of animals included in the studies. The limited diversity of cell lines and small sample sizes may affect the reliability of the findings and may complicate the assessment of efficacy, toxicity, and safety. Moreover, limited biological diversity may lead to the overestimation of efficacy, since nanotheranostic systems may appear effective in specific cell lines but fail to work in other cancer subtypes. Another limitation of these nanotheranostics may be their possible toxicity due to slow degradation when used in humans. Despite the fact that some of the studies demonstrated a very low or insignificant toxicity of nanotheranostic systems in vitro and in vivo, the mechanisms leading to the diffusion of nanotheranostics in animals and in humans are different. The next important limitation of these nanotheranostics is their low accumulation in tumors, which significantly reduces their efficacy. The success of cancer nanotheranostic systems largely depends on their ability to accumulate at tumor sites in sufficient concentrations, because effective accumulation ensures localized therapeutic effects and enhanced imaging contrast. However, despite the fact that the most of the evaluated nanotheranostic systems presented active targeting, they exhibited poor tumor accumulation, often less than 1%, with significant sequestration by the liver and the spleen. Furthermore, adequate knowledge about the kinetics of drug loading and release, nanocarrier degradation, pharmacokinetics, and the long-term impact of nanotheranostic systems on cellular signaling pathways and biochemical processes of the human body is absent or is still under study.

To accelerate progress in and the successful translation of nanotheranostic systems from bench to bedside, the integration of nanomedicine with AI should be sought to enhance the interpretation of complex molecular data. Such interdisciplinary research will require cross-disciplinary teams and collaboration between researchers, clinicians, engineers, and data scientists. Apart from these limitations of nanotheranostic systems, several important challenges also remain to be overcome before their successful translation from bench to bedside. One of them is batch-to-batch reproducibility. At the initial laboratory level, nanosystems are synthesized using different techniques, which vary significantly from case to case. Therefore, the suitable selection of materials and optimization of appropriate methods are necessary before their production. The second important challenge is scalability. This issue is multifaceted and includes difficulties with converting scientific findings into industrial technology, problems with the characteristics of nanomaterials, which alter when scaled up, and problems with the selection of materials and solvents [116]. One of the important aspects for scaling-up the production of nanoparticles/nanotheranostics is the cost of the final product and its acceptability for clinicians and patients.

To conclude, although a great effort is still needed for bench-to-bedside translation, interest in new innovative nanotheranostic systems will continue to grow in the future.

## Figures and Tables

**Table 1:** Summary of selected nanotheranostic systems for simultaneous cancer imaging and therapy. CTX—chemotherapy; RT—radiotherapy; PDT—photodynamic therapy; PTT—photothermal therapy; BNCT—boron neutron capture therapy; HIFU—high-intensity focused ultrasound; CDT—chemodynamic therapy; CL-PDT—chemiluminescence-mediated photodynamic therapy; MR-FUS—magnetic resonance-guided high-intensity focused ultrasound hyperthermia; MRI—magnetic resonance imaging; CT—computed tomography; USI—ultrasound imaging; SPECT—single-photon emission computed tomography; PET—positron imaging tomography; PAI—photoacoustic imaging; NIFR—near-infrared fluorescence imaging; FI—fluorescence imaging; FRET—fluorescence resonance energy transfer imaging; NPs—nanoparticles.

Type of NPs	Composition of Nanotheranostic System and Final Compound	NP Size (nm)	Therapeutic Modality	Imaging Modality	Cell Type/Animal Models	Ref.
iron-based NPs	Fe_3_O_4_ NPs coated with thermo-responsive fluorescent polymer and loaded with doxorubicin [Dox-TFP-MNPs]	~135	CTX	FI/MRI	human dermal fibroblasts (HDFs), normal prostate epithelial cells (PZ-HPV-7), human skin cancer A431 and G361 cells, human prostate cancer PC3 and LNCaP cells, NOD SCID mice bearing PC3-KD xenografts	[45]
	Fe_3_O_4_ NPs modified with alendronate-β-cyclodextrin conjugate and cross-linked by curcumin [CUR/ALN-β-CD-SPIONs]	200	CTX	MRI	Balb/c mice bearing murine 4T1 xenografts	[46]
	ferrite NPs modified with PEG and functionalized with YC-9 [YC-9-MNPs]	147 ± 8	PDT	FI/MRI	human prostate cancer PSMA(+) PC3 PIP and PSMA(−) PC3 flu cells, non-obese diabetic severe-combined immune deficient gamma (NSG) mice bearing PSMA(+) PC3 PIP or PSMA(−) PC3 flu xenografts	[47]
	Fe_3_O_4_ NPs loaded with Cy5.5 [Fe_3_O_4_-Cy5.5]	190	PTT	MRI/NIRF/PAI	rat glioma C6 cells, bone marrow macrophages, rats bearing C6 glioma xenografts	[48]
	Fe_3_O_4_ NPs coated with polycyclodextrin, functionalized with gadolinium ions and loaded with curcumin [CUR-Fe_3_O_4_@PCD-Gd]	57	CTX	MRI	human mammary epithelial MCF 10A cells, murine breast cancer 4T1 cells, BABL/c mice, Balb/c mice bearing murine 4T1 xenografts	[49]
	iron (II) tungstate NPs functionalized with hyaluronic acid [HA-FeWO 4 NPs]	91.4	PTT	MRI/CT	murine breast cancer 4T1 cells, human normal breast MCF-10A cells, Balb/c mice, Balb/c mice bearing murine 4T1 xenografts	[50]
gold NPs	Au nanorods coated with manganese dioxide nanosheet and cancer cell membrane and loaded with doxorubicin [CM-DOX-GMNPs]	164.5 ± 0.7	PTT/CTX	MRI/PTI	murine breast cancer 4T1 cells, BABL/c mice, Balb/c mice bearing murine 4T1 xenografts	[51]
	Au nanorods modified with poly(ethylene glycol) and coated with polydopamine [GNR@PDAs]	51.1 ± 4.2 × 7.2 ± 1.2	PTT	PAI	human ovarian adenocarcinoma SKOV3 cells	[52]
	Au NPs modified with poly(ethylene glycol) and DOTA and functionalized with bombesin analog (BBN [7,8,9,10,11,12,13,14]) and a Pt(IV) and labeled with ^67^Ga [^67^Ga-AuNP-BBN-Pt1]	104.1–112.5	CTX	SPECT	human prostate cancer PC3 cells, non-tumoral prostate RWPE-1 cells, Balb/c-Nude mice bearing PC3 xenografts	[53]
	Au NPs modified with L-Cys-DTPA ligand and propargylamine crosslinker and labeled with AlexaFluor 647 [Au(L-Cys DTPA) (Propargylamine)-AF647]	1–2	RT	FI	rat 9LGS gliosarcoma cells	[54]
polymeric NPs	NIR absorbing semiconducting polymer NPs with iodine-grafted amphiphilic copolymer [SNP-1]	20–30	PDT	FI/CT	Lewis lung carcinoma cells, c57bl/6 mice bearing Lewis cell xenografts	[55]
	polymer NPs composed of polysaccharide sodium alginate, modified with caspase 3 enzyme responsive activatable imaging probe and loaded with β- cyclodextrin-adamantane complex with kinase inhibitor [SPNs]	200	therapy by inhibition of PI3K/mTOR signaling pathway	FRET	murine BRAFV600E melanoma D4M cells, murine breast cancer 4T1 cells, C57B/L6 mice bearing D4M xenografts	[56]
	photothermal hybrid donor-acceptor polymer NPs modified with chitosan and functionalized with hyaluronic acid [HA-HDAPPs]	172–242	PTT	FI	murine colorectal carcinoma CT26.WT-Fluc-Neo (CT26) cells transduced with lentivirus encoded with firefly luciferase and a neomycin resistance gene, BALB/c mice bearing CT26.WT-Fluc-Neo (CT26) xenografts	[57]
	PLGA-b-PEG NPs functionalized with deferoxamine B and ACUPA, loaded with carborane and labeled with ^89^Zr [[^89^Zr]DFB(25)ACUPA(75)]	~160	BNCT	PET	human prostate adenocarcinoma PC3-flu and PC3-pip cells transfected with PSMA, nu/nu athymic mice bearing PC3-flu and PC3-pip xenografts	[58]
	polymer NPs loaded with gadolinium-porphyrin embedded in polyethyleneimine [Gd–PNPs]	200 nm	PDT	MRI/FI	human ovarian carcinoma Hela cells, murine colorectal carcinoma CT 26 cells, zebrafish animal model, BALB/c mice bearing murine CT26 xenografts	[59]
	polymeric NPs loaded with PTX-TIBA (2,3,5-Triiodobenzoic acid) prodrug [SS_TPTX_]	~81–103	CTX	CT	murine breast cancer 4T1 cells, Balb/c mice bearing murine 4T1 xenografts	[60]
	CuNC(Octa)-loaded PEG-PCL micelles [CuNC(Octa)-loaded micelles]	35–110	PTT	PAI	murine breast cancer 4T1 cells, Balb/c mice bearing 4T1 xenografts	[61]
	chitosan NPs loaded with palbociclib and ultra-small magnesium nanoclusters and functionalized with Folic acid and estrone [PB-UMN-CS-ES-FA-NPs]	198.2 ± 1.43	CTX	FI	human breast cancer MCF-7 cells, human breast cancer T-47D cells, SD rats with breast tumors induced by using 7,12 Dimethylbenzathracene (DMBA)	[62]
silica NPs	mesoporous organosilica NPs, doped with iron, modified with silane–PEG, functionalized with transferrin and loaded with doxorubicin [DOX@Fe-HMON-Tf]	71	CDT/CTX	MRI	human hepatoma cancer HepG2 cells, human fetal hepatocyte L-02 cells, Balb/c nude mice, bearing HepG2 xenografts	[63]
	mesoporous silica NPs modified with APTES, β-cyclodextrin and AMPPD and loaded with (4-carboxylphenyl) porphyrin (H6L) [MSN@H6L@β-CD@AMPPD NPs]	70	CL-PDT	NIRF	human normal liver HL-7702 cells, human hepatocarcinoma SMCC-7721 cells, human normal breast MCF-10A cells, murine breast cancer 4T1 cell line, BALB/c nude mice bearing SMCC-7721 xenografts	[64]
	mesoporous silica NPs decorated with graphene quantum dots and loaded with doxorubicin [DOX-carbanosilica]	120−140	PTA/CTX	NIFR	murine normal fibroblast L929 cells, murine breast cancer 4T1 cells, Balb/c mice bearing e 4T1 xenografts	[65]
	fibrous silica NPs modified with trimethoxysilyl-propyldiethylenetriamine and functionalized with chlorambucil, organotin metallodrug, folic acid and Alexa Fluor 647 [FS-DT-Chl-FA-Sn-AX]	427.9 ± 20.9	CTX	FI	human breast cancer MDA-MB-231 cells, NOD Scid IL2 receptor gamma chain KO mice bearing MDA-MB-231 xenografts	[66]
	silica-lipid NPs modified with poly(ethylene glycol) and loaded with iron oxides and perfluoropentane [SSPN]	289	HIFU	MRI/USI	human ovarian carcinoma Hela cells, BALB/c mice bearing CT26 xenografts	[67]
liposomes	DSCP (1,2-distearoyl-sn-glycero-3-phosphati dylcholine-[methoxy(polyethylene glycol)-2000) liposomes with indocyanine green J-aggregate, incorporated in lipid bilayer and loaded with doxorubicin [DOX-DSPC-IJA-HBS]	140–170	PTA/CTX	NIFR	BALB/c mice bearing CT 26 xenografts, BALB/c mice bearing 4T1 xenografts, NSG mice bearing C4-2B xenografts	[68]
	DiR-BOA-loaded HDL-like peptide- phospholipid NPs, functionalized with anti-TfR mAb, loaded with cholesterol-modified survivin siRNA [DiR-BOA-HPPS-mAb/siRNA]	29.26 ± 1.47	therapy by inhibition of the survivin expression at RNA level and induction of apoptosis	NIFR	human hepatic carcinoma HepG2 cells, human glioblastoma U87 cells, human cervical cancer HeLa cells, human breast cancer MDA-MB-231 cells, CHO-hTfR (human TfR) cells which stably express hTfR-GFP, control CHOvec cells, Balb/c nude mice bearing CHO-hTfR cells and CHOvec cells	[69]
	lecithin/cholesterol liposomes co-loaded with Au_4_Cu_4_ and Au_25_ nanoclusters [Au_4_Cu_4_ /Au_25_@Lip]	50	PTT/PDT	PTI/FI	human cervical cancer HeLa cells, Kunming mice bearing H22 xenografts	[70]
	imaging-thermosensitive liposomes conjugated with NIRF probe CF750.DSA, modified with DOTA and labeled with gadolinium-based contrast agents Gd.DOTA.DSA and loaded with doxorubicin [iTSL-DOX]	179 ± 3	MR-FUS/CTX	MRI/NIFR	human breast cancer MDA-MB-231 cells, CD-1 mice, athymic nude mice bearing MDA-MB-231 xenografts	[71]
dendrimers	G5 PAMAM dendrimers entrapped with gold nanoparticles and conjugated with nitroimidazole via a PEG linker and with Gd(III) by DOTA [Gd-Au-DENPs-Nit]	112.3	RT	MRI/CT	human nasopharyngeal carcinoma CNE-1 cell and human nasopharyngeal carcinoma hypoxia-resistant CNE-1H cells, normal murine NIH3T3 cells, BALB/c nude mice bearing CNE-1H xenografts	[72]
	PAMAM dendrimer-conjugated indocyanine green, bound to PEG-b-PCL polymer through a singlet oxygen-responsive thioketal bond and loaded with chlorin e6 (Ce6) [SNPICG/Ce6]	118 → 10	PTT/PDT	FI	murine breast cancer 4T1 cells, BALB/c nude mice bearing 4T1 xenografts	[73]
	G4 PAMAM dendrimers loaded with doxorubicin and copper sulfide and wrapped by amphiphilic gelatin [PRDCuS@AG]	200−250	PTT/CTX	PAI	murine fibroblast NIH-3T3 cells, murine sarcoma 4T1 cells, BALB/c nude mice bearing 4T1 xenografts	[74]
	G5 PAMAM dendrimers entrapped with CuS nanoparticles, modified with PEG, functionalized with RGD and 1,3-propane sultone and complexed with plasmid DNA-encoding pDNA-HIC1 [RGD-CuS DENPs/pDNA polyplexes]	157.9	PTT/gene therapy	PAI	human breast cancer MDA-MB-231 cells, BALB/c-nude mice bearing MDA-MB-231 xenografts	[75]
carbon-based NPs	graphene oxide (GO) nanosheets modified with poly(l-lysine), conjugated with Cit and G4-PAMAM dendrimers loaded with anti-miR-21 oligonucleotide and indocyanine green [GPCP/miR-21i/ICG]	~250	PTT/gene therapy	PTI/FI	human breast cancer MDA-MB-231 cells, BALB/c-nude mice bearing MDA-MB-231 xenografts	[76]
	carbon dots labeled with fluorescent 2-aminophenyl boronic acid and modified with chalcone [chalcone-APBA-CDs]	8.721	CTX	FI	human cervical cancer HeLa cells, fibrosarcoma cancer-bearing mice	[77]
	heparin-derived N, S-doped carbon dots, modified with polyethylenimine and loaded with baicalin [BA-PHCDs]	13 ± 2	CTX	FI	human lung cancer A549 cells, murine fibroblast NIH/3T3 cells	[78]
core–shell and composite NPs	core–shell Si-Au NPs loaded with PDT agent Ir1 [Ir1- AuSiO_2_]	40 ± 2	PTT/PDT	LI	athymic mice bearing human glioblastoma Gli36D5 xenografts	[79]
	2D titanium carbide MXene nanosheets, functionalized with core–shell NaErF_4_:0.5%Tm@NaLuF_4_ (NaErF_4_) nanoparticles [NaErF_4_@ Ti_3_C_2_]	200 × 4.5	PTT	MRI/FI	human hepatoma HepG2 cells, BALB/c-nu mice bearing HepG2 xenografts	[80]
	2D titanium carbide MXene nanosheets, decorated with platinum nanoparticles and modified with PEG [Ti_3_C_2_Tx-Pt-PEG]	200	PTT/enzyme therapy	PAI	murine breast cancer 4T1 cells, murine fibroblast L929 cells, BALB/c mice bearing 4T1 xenografts	[81]
	2D stimuli-responsive nanocomposites comprising graphene oxide nanosheets decorated with Au NPs and Fe_3_O_4_ NPs, coated with doxorubicin-loaded 1-tetradecanol, and modified with alginate polymer [GO-SPIO-Au-DOX-TD-Alg NCs]	40	PTT/CXT	MRI/CT	murine colon adenocarcinoma CT26 cells, Balb/c mice bearing CT 26 xenografts	[82]
	copper sulfide NPs, coated with polyethyleneimine, loaded with indocyanine green and functionalized with folic acid [CuS-PEI-ICG-FA]	<10	PTT	PAI	human cervical cancer HeLa cells, Balb/c mice bearing HeLa xenografts	[34]
	Au- Fe_3_O_4_ Janus NPs modified with DHCA and functionalized with RGD [GION@RGD]	18.7	PTT/therapy by NIFR-enhanced ferroptosis	MRI	murine breast cancer 4T1 cells, Balb/c nude mice bearing 4T1 xenografts	[83]

## Data Availability

The data presented in this study are available in the Supplementary Material.

## Supplementary Materials

The following supporting information can be downloaded at https://www.mdpi.com/article/10.3390/molecules29245985/s1, Table S1: The advantages and limitations of imaging modalities.
