# Supplementary material for: Advances in Nanotheranostic Systems for Concurrent Cancer Imaging and Therapy: An Overview of the Last 5 Years
Source: Molecules. 2024 Dec 19;29(24):5985. doi: 10.3390/molecules29245985 (PMC11677634; doi:10.3390/molecules29245985)
Supplement: Supplementary file 1 [file molecules-29-05985-s001.zip › molecules-3334748-supplementary.pdf]

Supplementary Table S1.
The advantages and limitations of imaging modalities

Imaging modality	Spatial resolution	Advantages	Disadvantages
MRI	~ 25 µm-1 mm	excellent spatial resolution and depth of penetration in soft tissues, structural and functional data, great contrast for soft tissues	long acquisition times (~minutes - hours), overheating or malfunction of metal implants in the body, possible reactions to the contrast agent
USI	~ 50–500 µm	low cost, no radiation, high speed, portable,	low contrast, variable spatial resolution, lower than MRI and CT, can struggle with fine detail, especially in deeper structures due to attenuation
CT	~ 50 µm	high spatial resolution, particularly for dense structures like bones	low contrast, possible mild to severe allergic reaction or kidney damage due to the contrast agent, exposure to ionizing radiation
SPECT	~ 1–5 mm	lower cost, longer half-life of radio tracers and longer imaging time, compared to PET	limited spatial resolution, exposure to ionizing radiation
PET	~ 1-5 mm	high image quality, interpretive certainty, diagnostic accuracy, low patient dosimetry and short acquisition times (~ 30 min.)	high-cost compared to SPECT, limited imaging time window, possibility of false positive diagnosis, not widely available, exposure to ionizing radiation
PAI	~ 10 µm to 1 mm	no radiation, high spatial resolution, anatomical, functional and molecular imaging, lower cost compared to CT and PET	temperature dependent signal, weak absorption at shorter wavelengths, distortion of acoustic signal due to skull
FI	~few mm – few cm	no radiation, wealth of molecular and structural information	poor spatial resolution and limited depth of penetration (~few cm) in tissue, photobleaching
NIR-I	~ 100 µm to 1 mm	approved by the FDA for clinical diagnosis (indocyanine green), high temporal and spatial resolution	limited to cell and tissue samples with low thickness, high background, interfering with imaging, low safety threshold of excitation light
NIR-II	~ 10 µm	higher temporal resolution, spatial resolution, signal-to-background ratio and stronger penetrability of deep tissue, compared to NIR-I, minimal autofluorescence,	high absorption by water, light attenuation in tissue and blood, high cost of instrumentation with optimal performance,
FTIR	~ 5–10 µm	high sensitivity, real-time measurements, processing and display simultaneous quantification of many compounds, no radiation, label-free, short imaging time	poor distinction of closely related molecular structures, high attenuation in liquid environment
